# Supplementary material for: Treatment strategies for stage IA non-small cell lung cancer: A SEER-based population study
Source: PLoS One. 2024 Apr 29;19(4):e0298470. doi: 10.1371/journal.pone.0298470 (PMC11057715; doi:10.1371/journal.pone.0298470)

Supplementary Data – Figures

Figure S1 Kaplan–Meier curves of OS (**A**) and LCSS (**B**) and HR (95% CI) of OS (**C**) and LCSS (**D**) among multiple treatment modalities in patients with marital status (married).

Figure S2 Kaplan–Meier curves of OS (**A**) and LCSS (**B**) and HR (95% CI) of OS (**C**) and LCSS (**D**) among multiple treatment modalities in patients with years of diagnosis (2014-2018).

Figure S3 Kaplan–Meier curves of OS (**A**) and LCSS (**B**) and HR (95% CI) of OS (**C**) and LCSS (**D**) among multiple treatment modalities in patients with histologic type (others).

Figure S4 Kaplan–Meier curves of OS (**A**) and LCSS (**B**) and HR (95% CI) of OS (**C**) and LCSS (**D**) among multiple treatment modalities in patients with location (middle lobe).

Figure S5 Kaplan–Meier curves of OS (**A**) and LCSS (**B**) and HR (95% CI) of OS (**C**) and LCSS (**D**) among multiple treatment modalities in patients with ethnicity (black).

Figure S6 Kaplan–Meier curves of OS (**A**) and LCSS (**B**) and HR (95% CI) of OS (**C**) and LCSS (**D**) among multiple treatment modalities in patients with histologic type (LSCC).

Figure S7 Kaplan–Meier curves of OS (**A**) and LCSS (**B**) and HR (95% CI) of OS (**C**) and LCSS (**D**) among multiple treatment modalities in patients with ethnicity (Asian or Pacific Islander).

Figure S8 Kaplan–Meier curves of OS (**A**) and LCSS (**B**) and HR (95% CI) of OS (**C**)

and LCSS (**D**) among multiple treatment modalities in patients with histologic type (LADC).

Figure S9 Kaplan–Meier curves of OS (**A**) and LCSS (**B**) and HR (95% CI) of OS (**C**) and LCSS (**D**) among multiple treatment modalities in patients with years of diagnosis (2004-2008).

Figure S10 Kaplan–Meier curves of OS (**A**) and LCSS (**B**) and HR (95% CI) of OS (**C**) and LCSS (**D**) among multiple treatment modalities in patients with years of diagnosis (2009-2013).

Figure S11 Kaplan–Meier curves of OS (**A**) and LCSS (**B**) and HR (95% CI) of OS (**C**) and LCSS (**D**) among multiple treatment modalities in patients with sex (male).

Figure S12 Kaplan–Meier curves of OS (**A**) and LCSS (**B**) and HR (95% CI) of OS (**C**) and LCSS (**D**) among multiple treatment modalities in patients with sex (female).

Figure S13 Kaplan–Meier curves of OS (**A**) and LCSS (**B**) and HR (95% CI) of OS (**C**) and LCSS (**D**) among multiple treatment modalities in patients with age (>65 years).

Figure S14 Kaplan–Meier curves of OS (**A**) and LCSS (**B**) and HR (95% CI) of OS (**C**) and LCSS (**D**) among multiple treatment modalities in patients with location (upper lobe).

Figure S15 Kaplan–Meier curves of OS (**A**) and LCSS (**B**) and HR (95% CI) of OS (**C**) and LCSS (**D**) among multiple treatment modalities in patients with location (lower lobe).

Figure S16 Kaplan–Meier curves of OS (**A**) and LCSS (**B**) and HR (95% CI) of OS

(**C**) and LCSS (**D**) among multiple treatment modalities in patients with marital status (single).

Figure S17 Kaplan–Meier curves of OS (**A**) and LCSS (**B**) and HR (95% CI) of OS (**C**) and LCSS (**D**) among multiple treatment modalities in patients with ethnicity (white).

Figure S18 Kaplan–Meier curves of OS (**A**) and LCSS (**B**) and HR (95% CI) of OS (**C**) and LCSS (**D**) among different surgery modalities in patients with age (≤65 years).

Figure S19 Kaplan–Meier curves of OS (**A**) and LCSS (**B**) and HR (95% CI) of OS (**C**) and LCSS (**D**) among different surgery modalities in patients with age (>65 years).

Figure S20 Kaplan–Meier curves of OS (**A**) and LCSS (**B**) and HR (95% CI) of OS (**C**) and LCSS (**D**) among different surgery modalities in patients with sex (male).

Figure S21 Kaplan–Meier curves of OS (**A**) and LCSS (**B**) and HR (95% CI) of OS (**C**) and LCSS (**D**) among different surgery modalities in patients with sex (female).

Figure S22 Kaplan–Meier curves of OS (**A**) and LCSS (**B**) and HR (95% CI) of OS (**C**) and LCSS (**D**) among different surgery modalities in patients with ethnicity (white).

Figure S23 Kaplan–Meier curves of OS (**A**) and LCSS (**B**) and HR (95% CI) of OS (**C**) and LCSS (**D**) among different surgery modalities in patients with ethnicity (black).

Figure S24 Kaplan–Meier curves of OS (**A**) and LCSS (**B**) and HR (95% CI) of OS

(**C**) and LCSS (**D**) among different surgery modalities in patients with ethnicity (Asian or Pacific Islander).

Figure S25 Kaplan–Meier curves of OS (**A**) and LCSS (**B**) and HR (95% CI) of OS (**C**) and LCSS (**D**) among different surgery modalities in patients with years of diagnosis (2004-2008).

Figure S26 Kaplan–Meier curves of OS (**A**) and LCSS (**B**) and HR (95% CI) of OS (**C**) and LCSS (**D**) among different surgery modalities in patients with years of diagnosis (2009-2013).

Figure S27 Kaplan–Meier curves of OS (**A**) and LCSS (**B**) and HR (95% CI) of OS (**C**) and LCSS (**D**) among different surgery modalities in patients with years of diagnosis (2014-2018).

Figure S28 Kaplan–Meier curves of OS (**A**) and LCSS (**B**) and HR (95% CI) of OS (**C**) and LCSS (**D**) among different surgery modalities in patients with histologic type (LADC).

Figure S29 Kaplan–Meier curves of OS (**A**) and LCSS (**B**) and HR (95% CI) of OS (**C**) and LCSS (**D**) among different surgery modalities in patients with histologic type (LSCC).

Figure S30 Kaplan–Meier curves of OS (**A**) and LCSS (**B**) and HR (95% CI) of OS (**C**) and LCSS (**D**) among different surgery modalities in patients with histologic type (others).

Figure S31 Kaplan–Meier curves of OS (**A**) and LCSS (**B**) and HR (95% CI) of OS (**C**) and LCSS (**D**) among different surgery modalities in patients with location (upper

lobe).

Figure S32 Kaplan–Meier curves of OS (**A**) and LCSS (**B**) and HR (95% CI) of OS (**C**) and LCSS (**D**) among different surgery modalities in patients with location (middle lobe).

Figure S33 Kaplan–Meier curves of OS (**A**) and LCSS (**B**) and HR (95% CI) of OS (**C**) and LCSS (**D**) among different surgery modalities in patients with location (lower lobe).

Figure S34 Kaplan–Meier curves of OS (**A**) and LCSS (**B**) and HR (95% CI) of OS (**C**) and LCSS (**D**) among different surgery modalities in patients with marital status (married).

Figure S35 Kaplan–Meier curves of OS (**A**) and LCSS (B) and HR (95% CI) of OS (**C**) and LCSS (**D**) among different surgery modalities in patients with marital status (single).

Figure S1 Kaplan–Meier curves of OS **(A)** and LCSS **(B)** and HR (95% CI) of OS **(C)** and LCSS **(D)** among multiple treatment modalities in patients with with marital status (married).

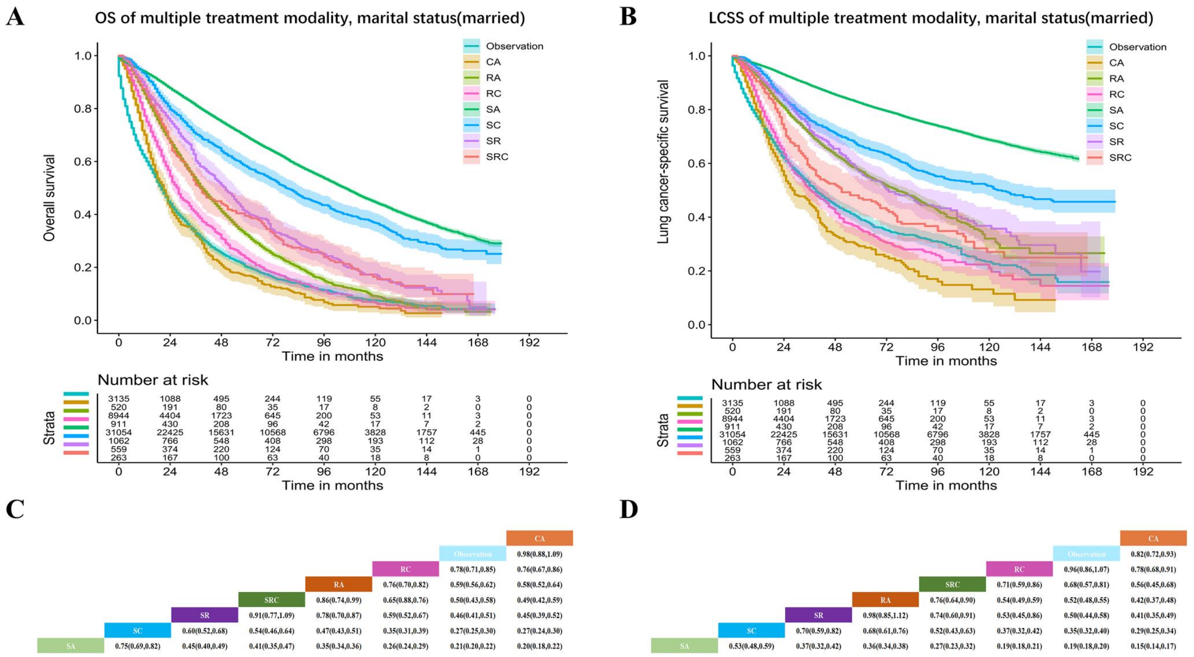

Figure S2 Kaplan–Meier curves of OS **(A)** and LCSS **(B)** and HR (95% CI) of OS **(C)** and LCSS **(D)** among multiple treatment modalities in patients with years of diagnosis (2014-2018).

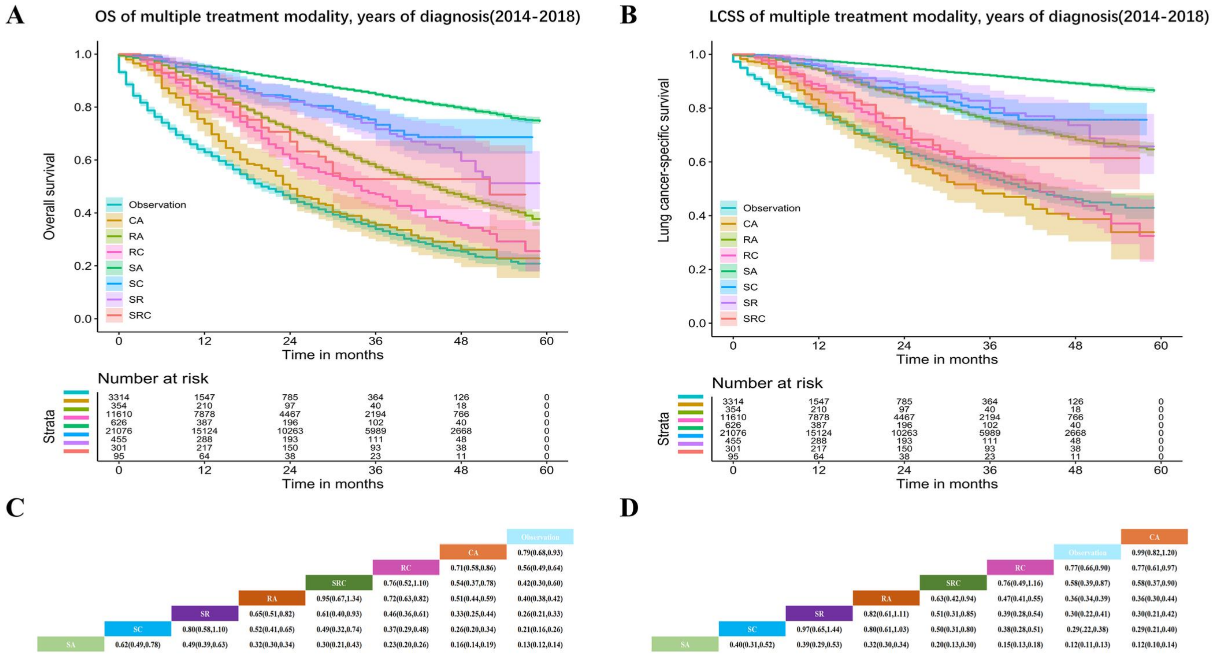

Figure S3 Kaplan–Meier curves of OS **(A)** and LCSS **(B)** and HR (95% CI) of OS **(C)** and LCSS **(D)** among multiple treatment modalities in patients with histologic type (others).

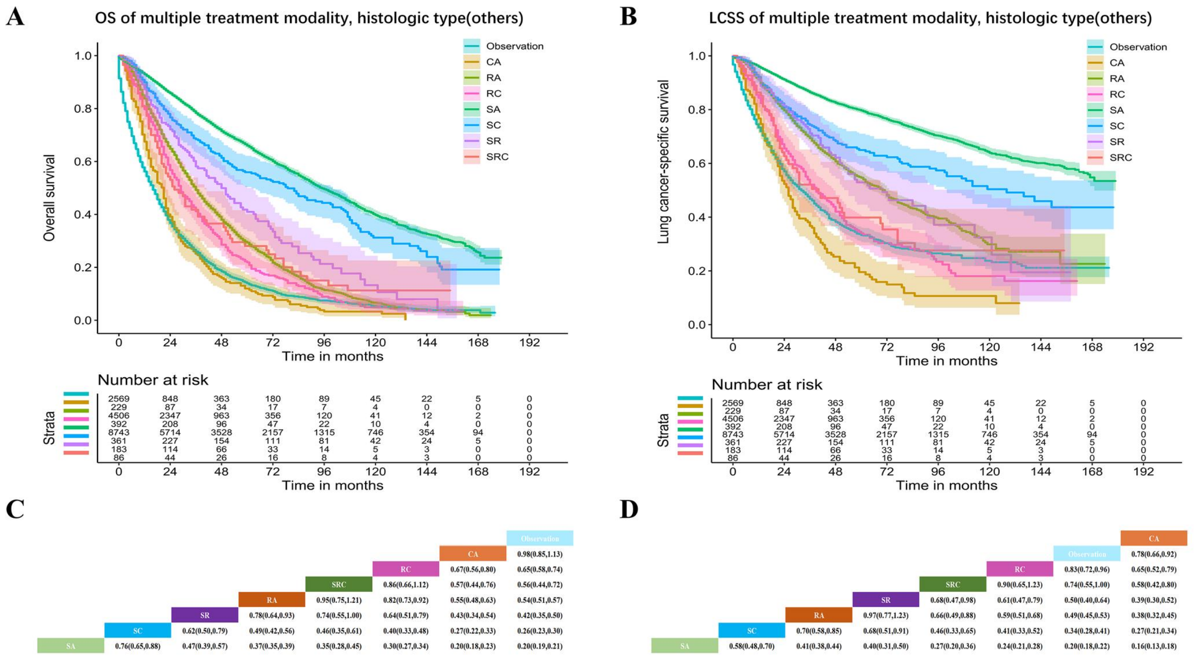

Figure S4 Kaplan–Meier curves of OS **(A)** and LCSS **(B)** and HR (95% CI) of OS **(C)** and LCSS **(D)** among multiple treatment modalities in patients with location (middle lobe).

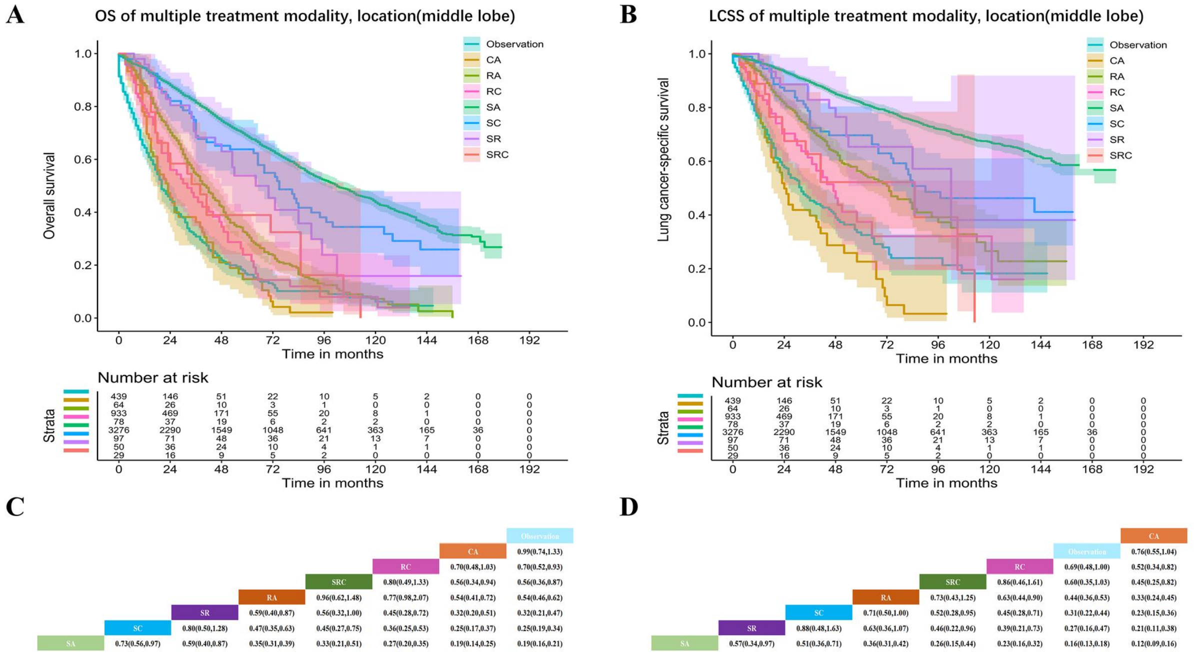

Figure S5 Kaplan–Meier curves of OS **(A)** and LCSS **(B)** and HR (95% CI) of OS **(C)** and LCSS **(D)** among multiple treatment modalities in patients with ethnicity (black).

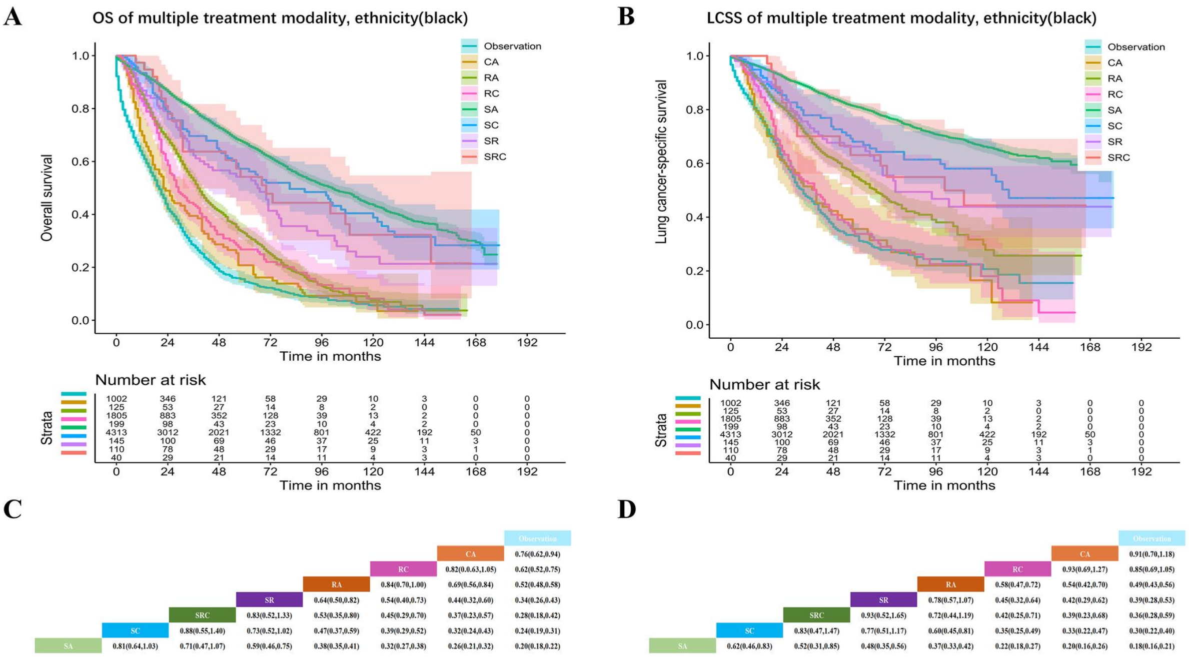

Figure S6 Kaplan–Meier curves of OS **(A)** and LCSS **(B)** and HR (95% CI) of OS **(C)** and LCSS **(D)** among multiple treatment modalities in patients with histologic type (LSCC).

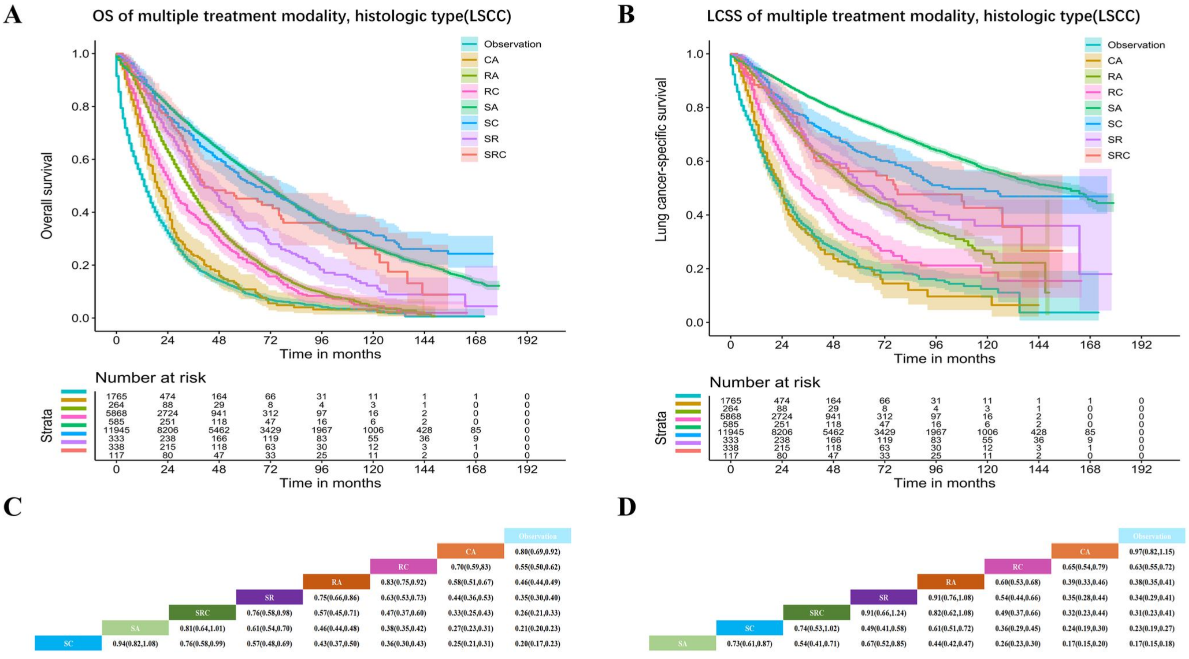

Figure S7 Kaplan–Meier curves of OS **(A)** and LCSS **(B)** and HR (95% CI) of OS **(C)** and LCSS **(D)** among multiple treatment modalities in patients with ethnicity (Asian or Pacific Islander).

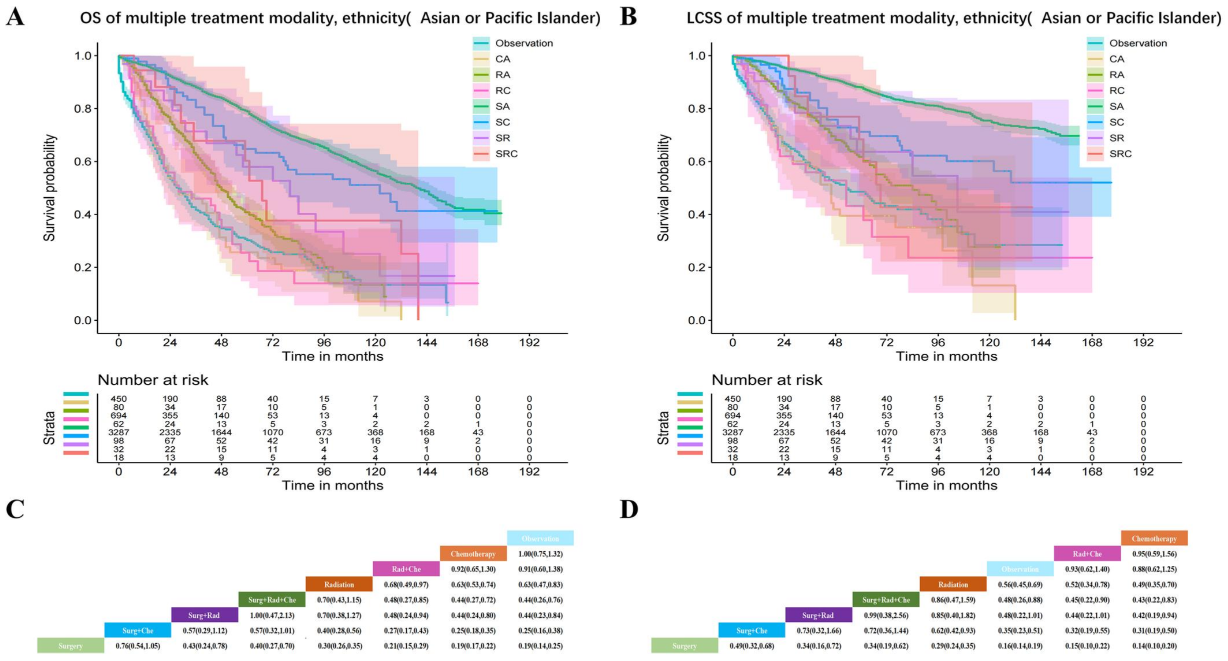

Figure S8 Kaplan–Meier curves of OS **(A)** and LCSS **(B)** and HR (95% CI) of OS **(C)** and LCSS **(D)** among multiple treatment modalities in patients with histologic type (LADC).

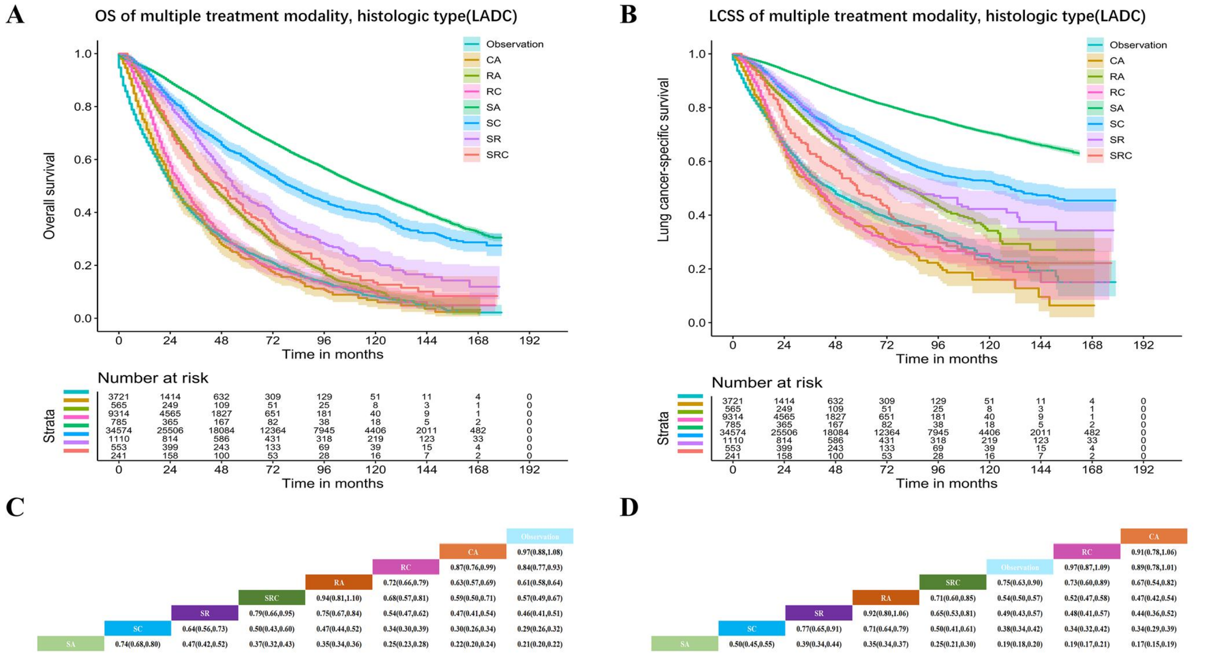

Figure S9 Kaplan–Meier curves of OS **(A)** and LCSS **(B)** and HR (95% CI) of OS **(C)** and LCSS **(D)** among multiple treatment modalities in patients with years of diagnosis (2004-2008).

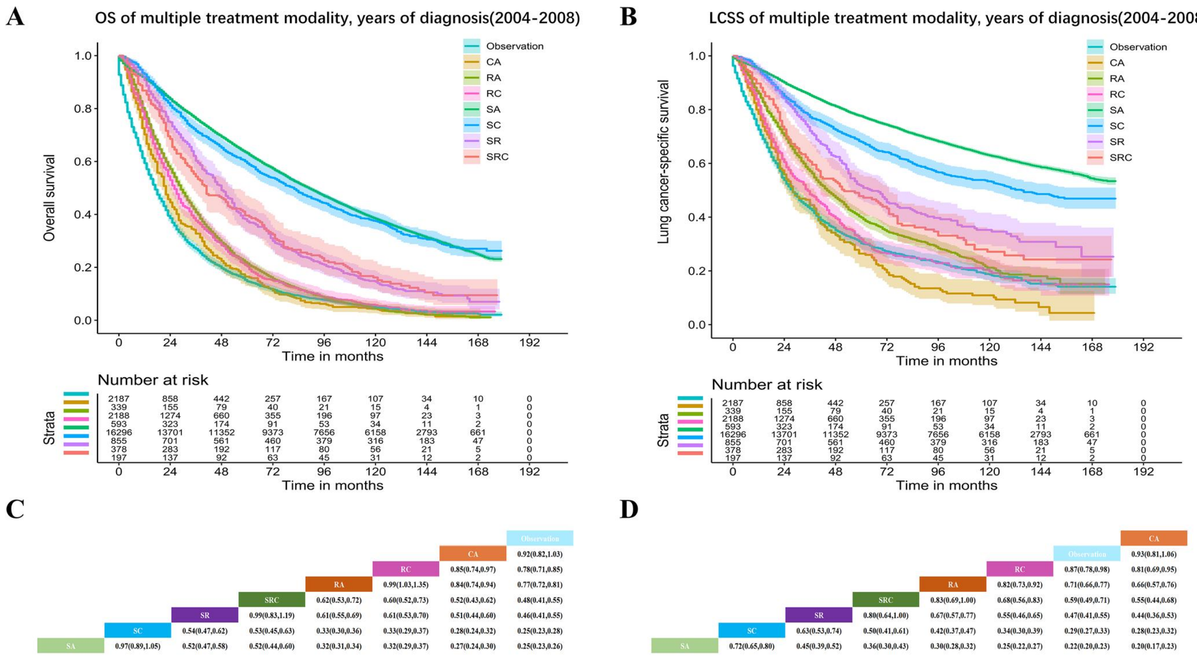

Figure S10 Kaplan–Meier curves of OS **(A)** and LCSS **(B)** and HR (95% CI) of OS **(C)** and LCSS **(D)** among multiple treatment modalities in patients with years of diagnosis (2009-2013).

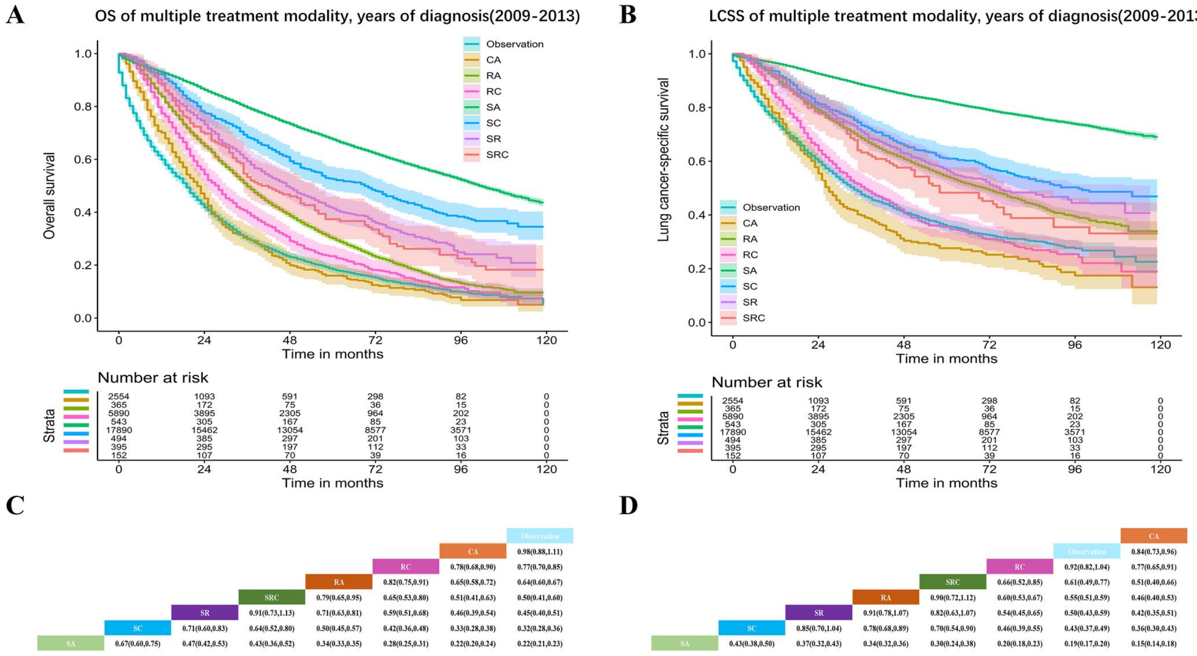

Figure S11 Kaplan–Meier curves of OS **(A)** and LCSS **(B)** and HR (95% CI) of OS **(C)** and LCSS **(D)** among multiple treatment modalities in patients with sex (male).

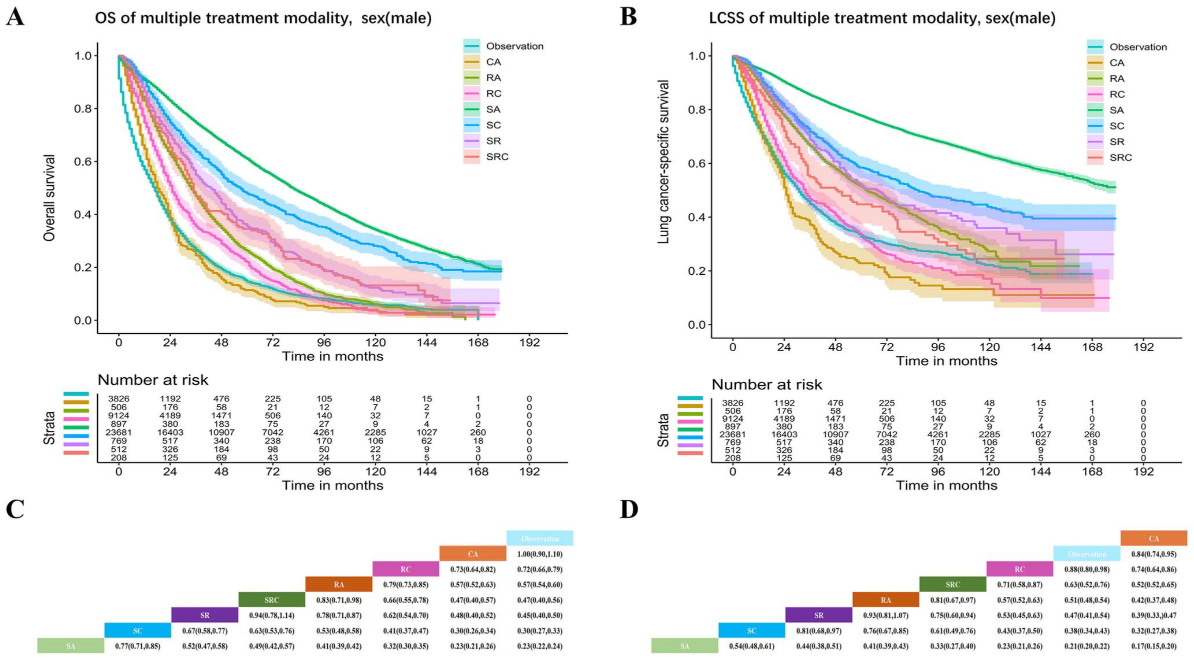

Figure S12 Kaplan–Meier curves of OS **(A)** and LCSS **(B)** and HR (95% CI) of OS **(C)** and LCSS **(D)** among multiple treatment modalities in patients with sex (female).

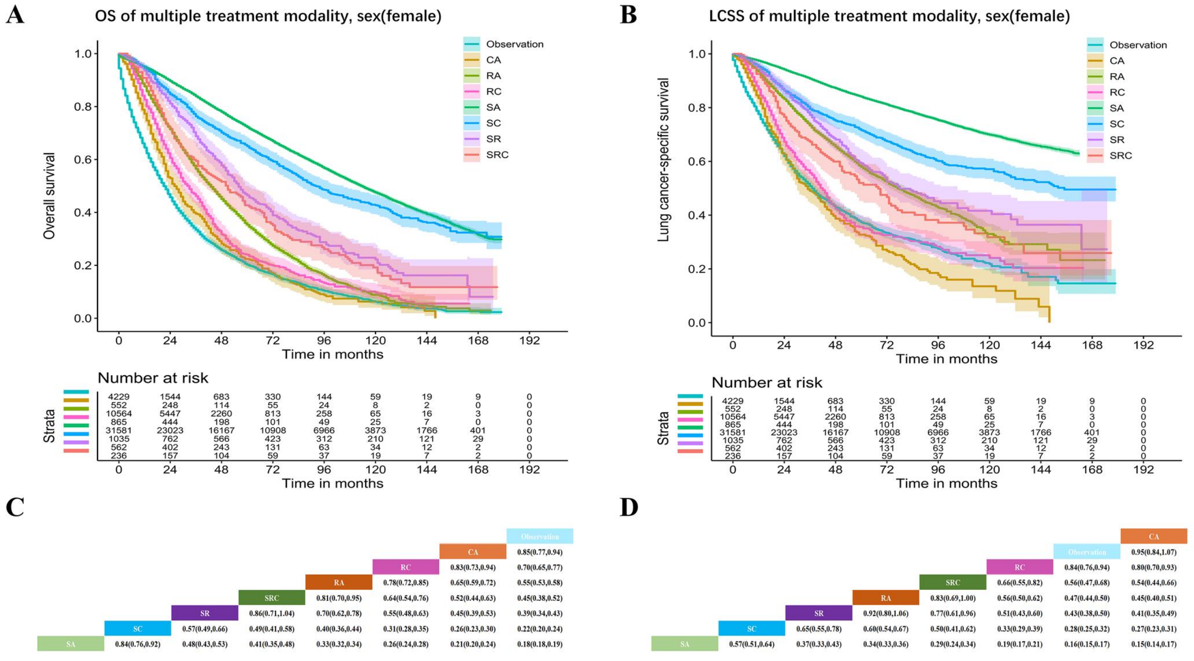

Figure S13 Kaplan–Meier curves of OS **(A)** and LCSS **(B)** and HR (95% CI) of OS **(C)** and LCSS **(D)** among multiple treatment modalities in patients with age (>65 years).

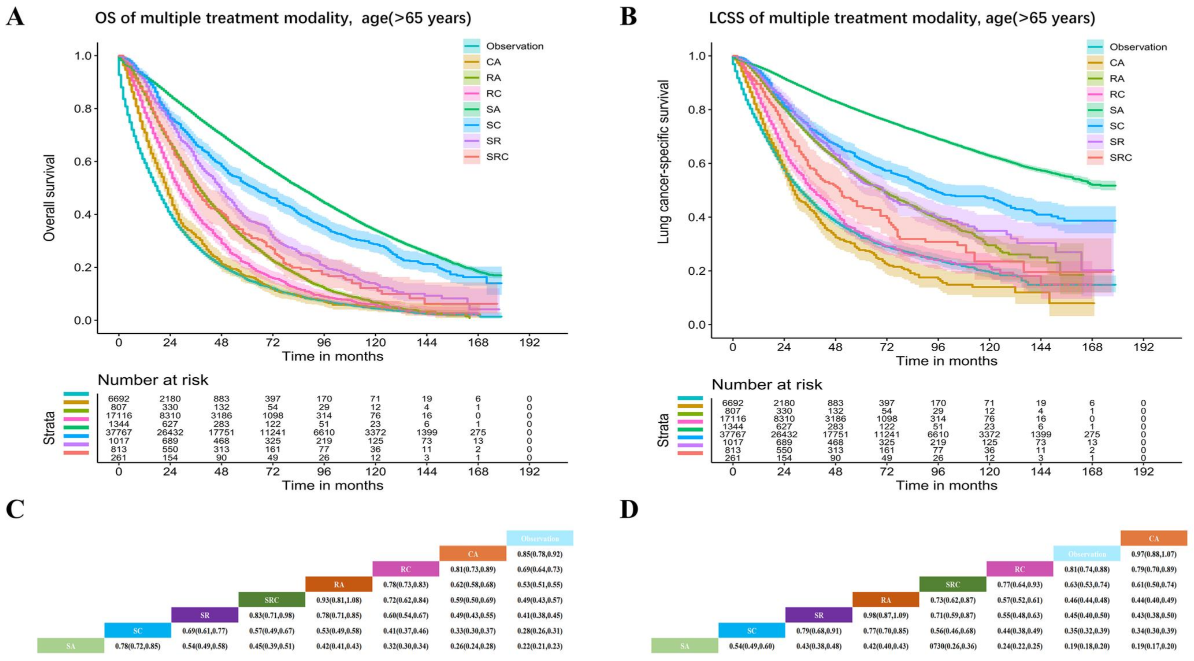

Figure S14 Kaplan–Meier curves of OS **(A)** and LCSS **(B)** and HR (95% CI) of OS **(C)** and LCSS **(D)** among multiple treatment modalities in patients with location (upper lobe).

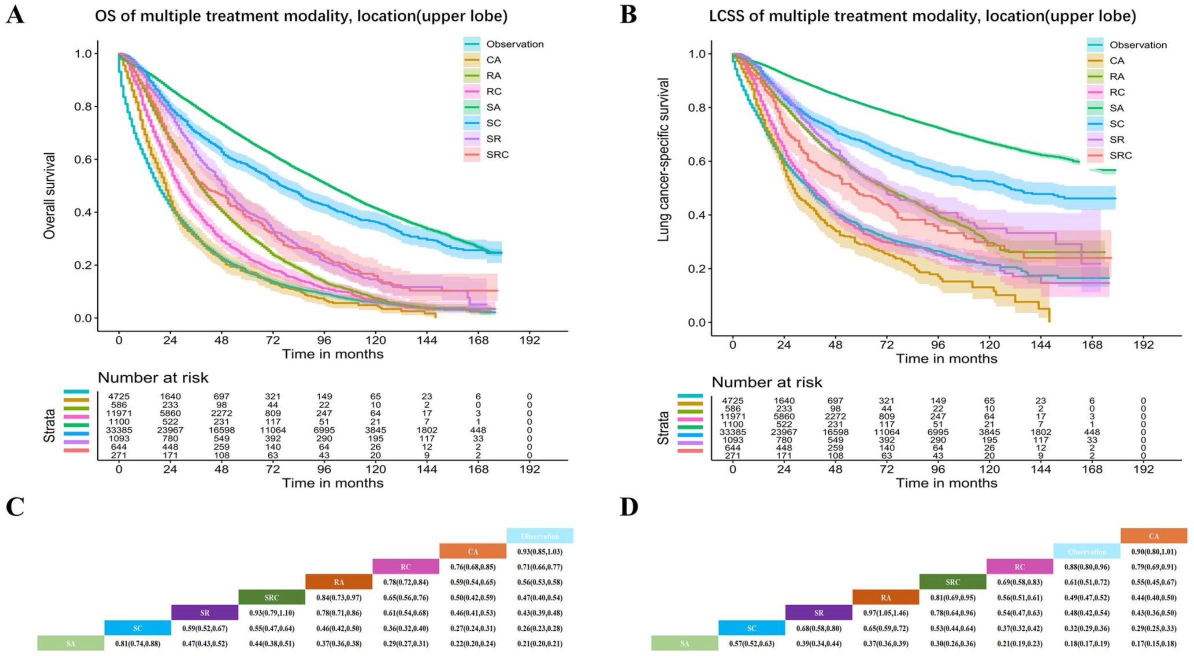

Figure S15 Kaplan–Meier curves of OS **(A)** and LCSS **(B)** and HR (95% CI) of OS **(C)** and LCSS **(D)** among multiple treatment modalities in patients with location (lower lobe).

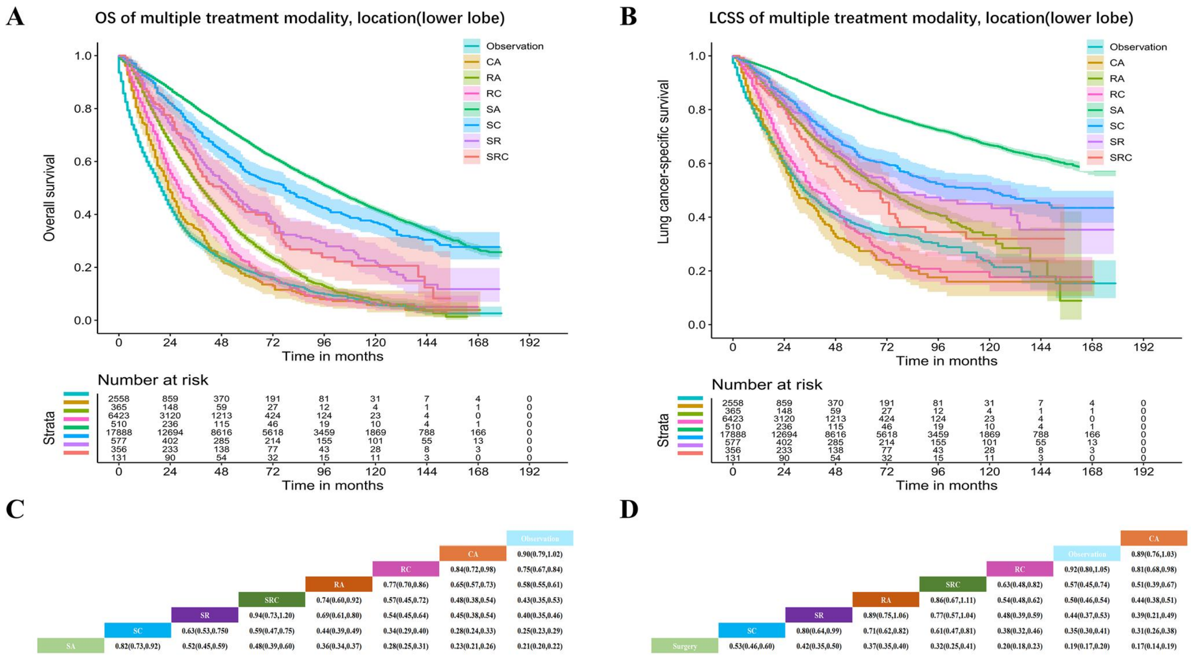

Figure S16 Kaplan–Meier curves of OS **(A)** and LCSS **(B)** and HR (95% CI) of OS **(C)** and LCSS **(D)** among multiple treatment modalities in patients with marital status (single).

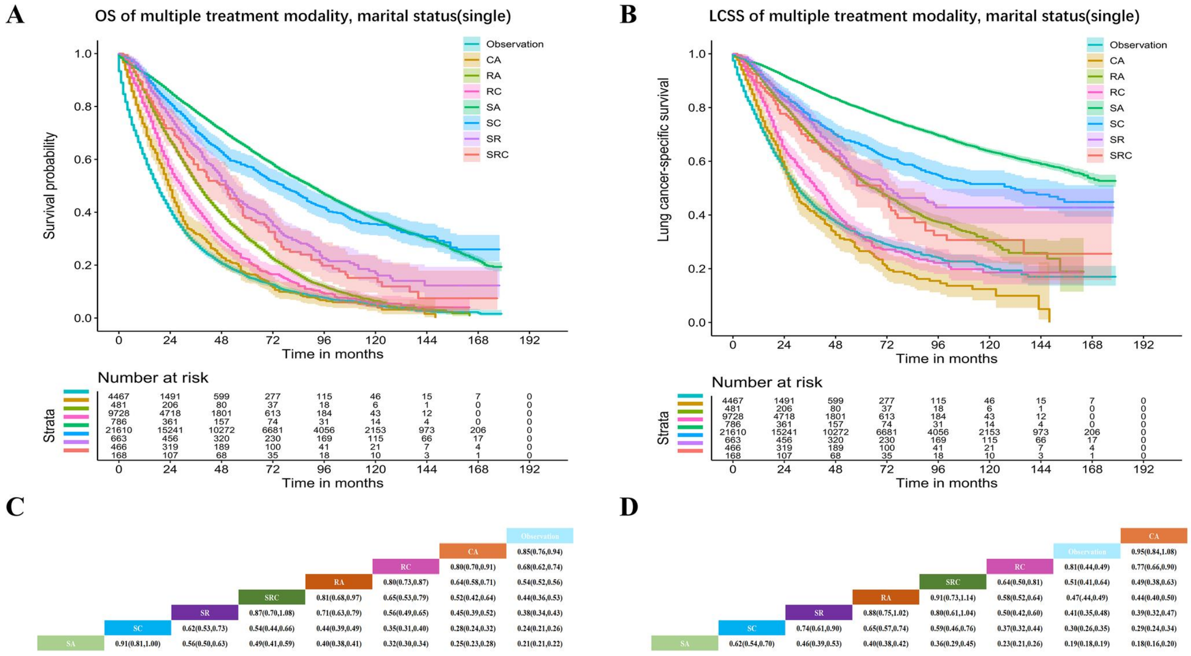

Figure S17 Kaplan–Meier curves of OS **(A)** and LCSS **(B)** and HR (95% CI) of OS **(C)** and LCSS **(D)** among multiple treatment modalities in patients with ethnicity (white).

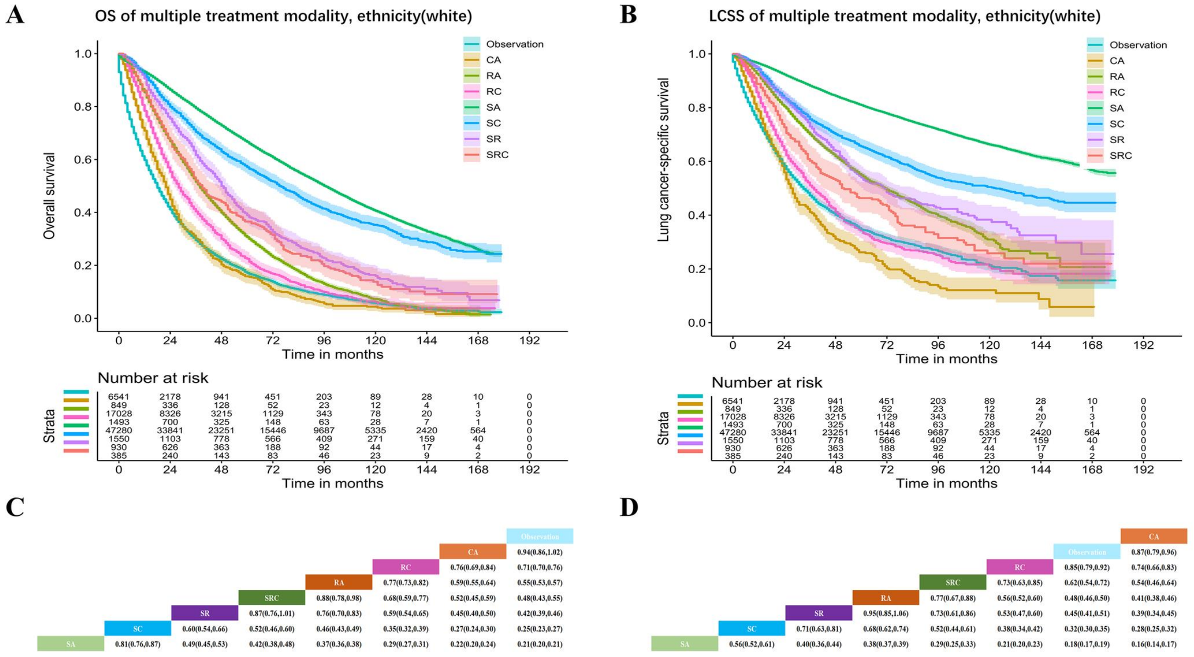

Figure S18 Kaplan–Meier curves of OS **(A)** and LCSS **(B)** and HR (95% CI) of OS **(C)** and LCSS **(D)** among different surgery modalities in patients with age (≤65 years).

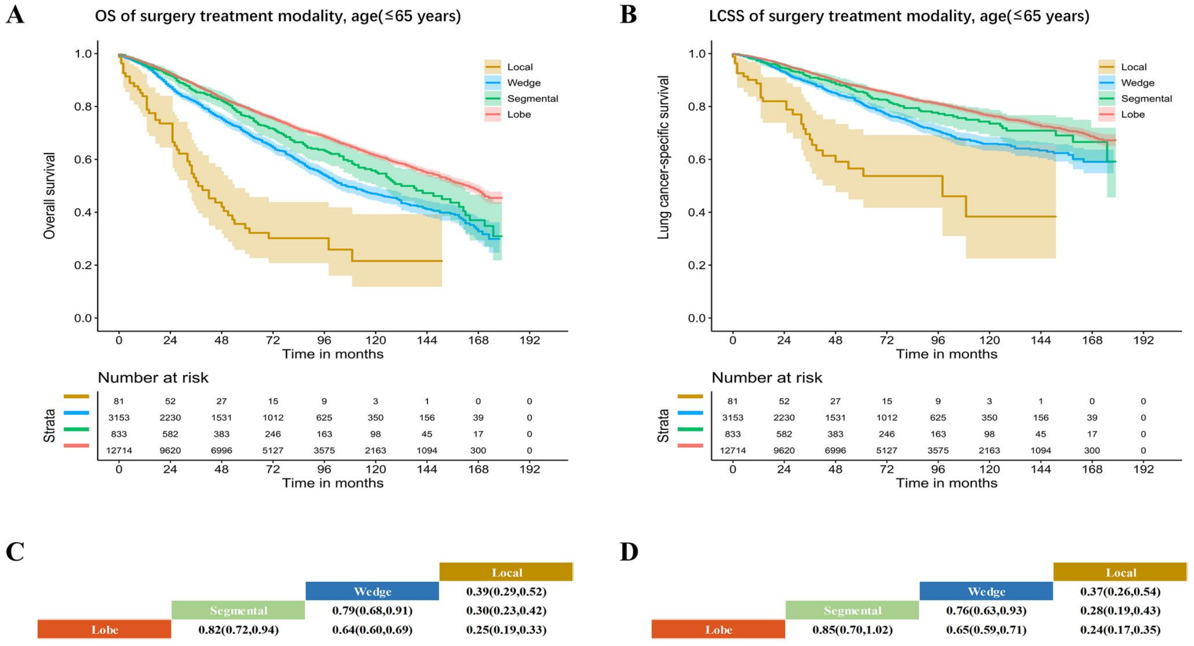

Figure S19 Kaplan–Meier curves of OS **(A)** and LCSS **(B)** and HR (95% CI) of OS **(C)** and LCSS **(D)** among different surgery modalities in patients with age (>65 years).

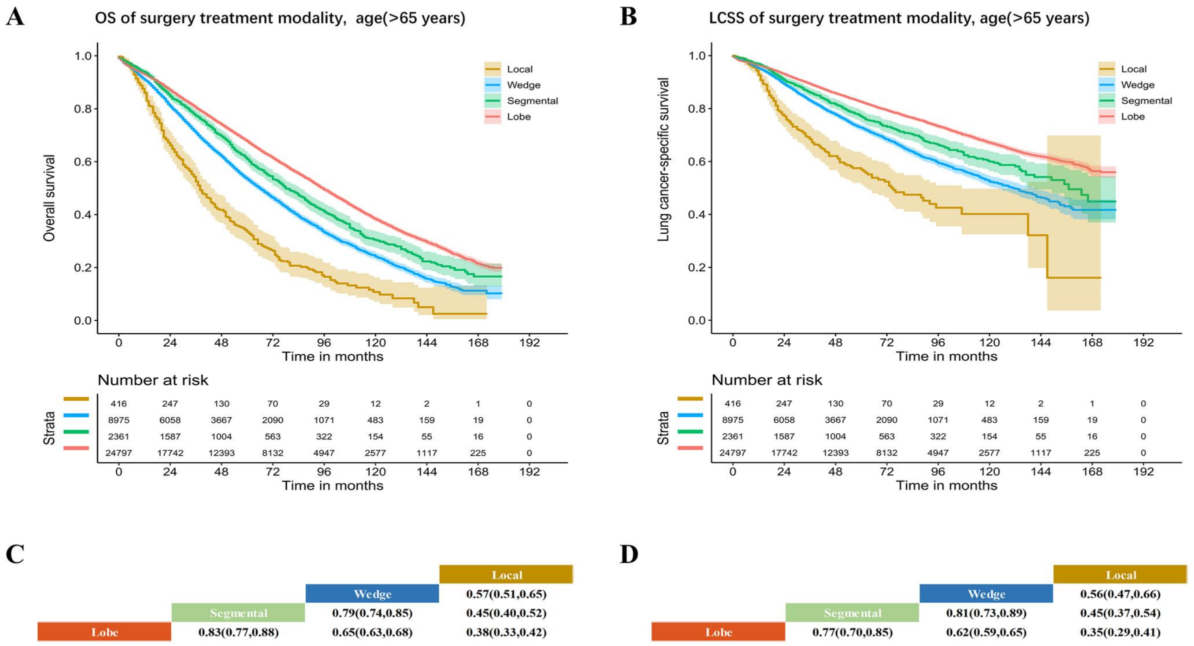

Figure S20 Kaplan–Meier curves of OS **(A)** and LCSS **(B)** and HR (95% CI) of OS **(C)** and LCSS **(D)** among different surgery modalities in patients with sex (male).

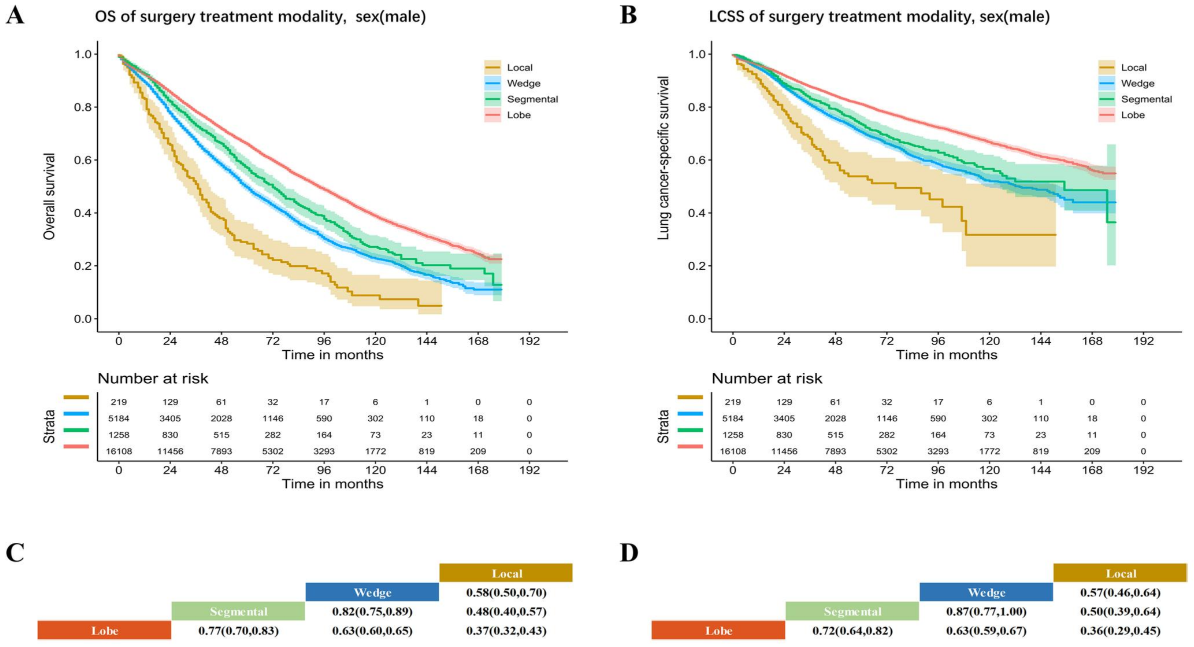

Figure S21 Kaplan–Meier curves of OS **(A)** and LCSS **(B)** and HR (95% CI) of OS **(C)** and LCSS **(D)** among different surgery modalities in patients with sex (female).

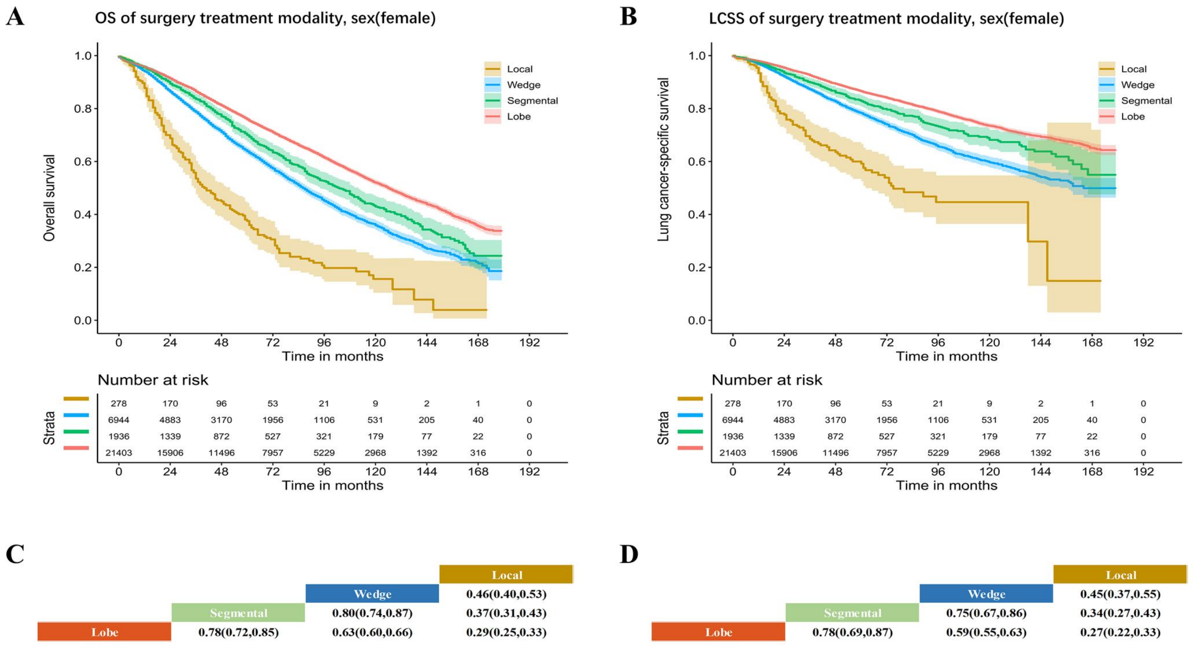

Figure S22 Kaplan–Meier curves of OS **(A)** and LCSS **(B)** and HR (95% CI) of OS **(C)** and LCSS **(D)** among different surgery modalities in patients with ethnicity (white).

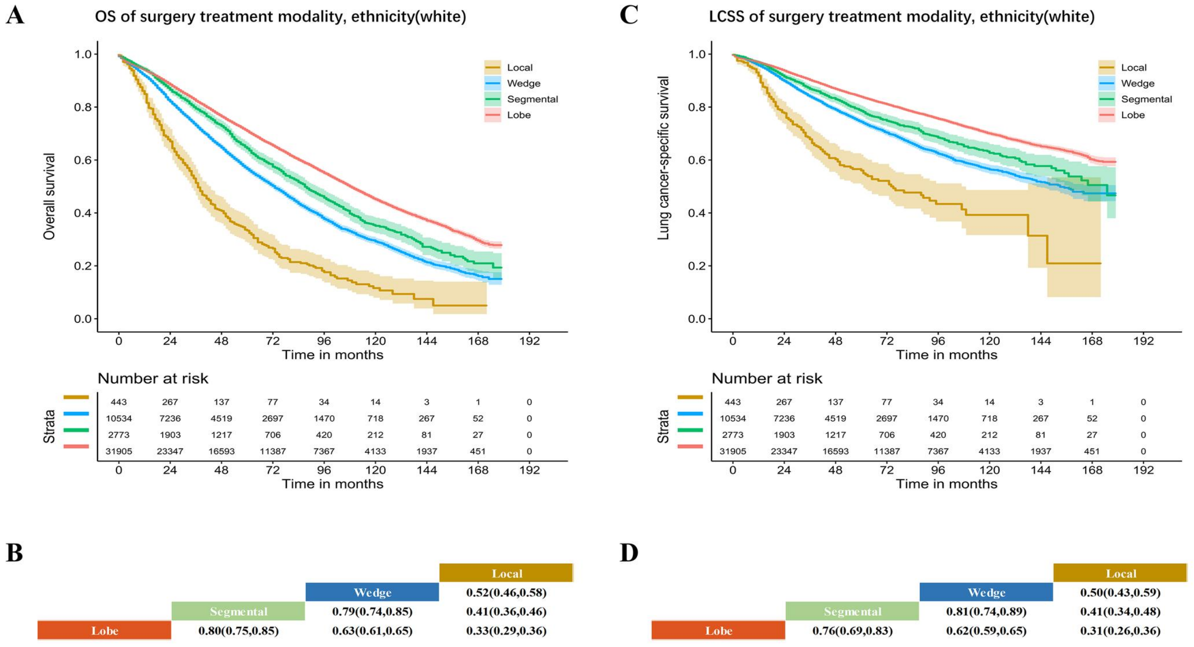

Figure S23 Kaplan–Meier curves of OS **(A)** and LCSS **(B)** and HR (95% CI) of OS **(C)** and LCSS **(D)** among different surgery modalities in patients with ethnicity (black).

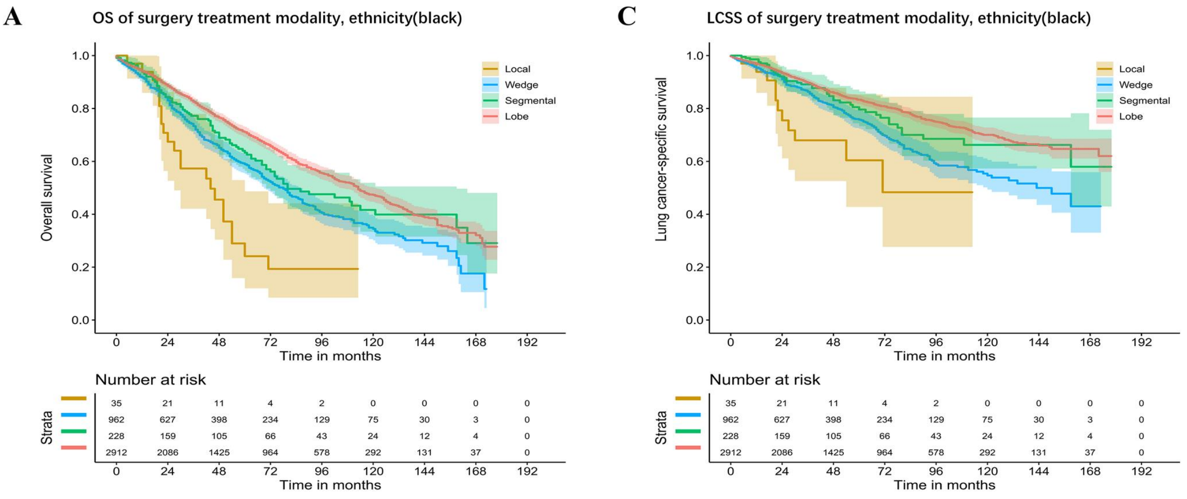

B

			Local
		Wedge	0.52(0.34,0.80)
	Segmental	0.82(0.65,1.03)	0.42(0.27,0.68)
Lobe	0.82(0.66,1.02)	0.67(0.60,0.76)	0.35(0.23,0.53)

D

			Local
		Wedge	0.58(0.32,1.06)
	Segmental	0.73(0.53,1.01)	0.42(0.22,0.82)
Lobe	0.86(0.63,1.17)	0.62(0.53,0.73)	0.36(0.20,0.66)

Figure S24 Kaplan–Meier curves of OS **(A)** and LCSS **(B)** and HR (95% CI) of OS **(C)** and LCSS **(D)** among different surgery modalities in patients ethnicity (Asian or Pacific Islander).

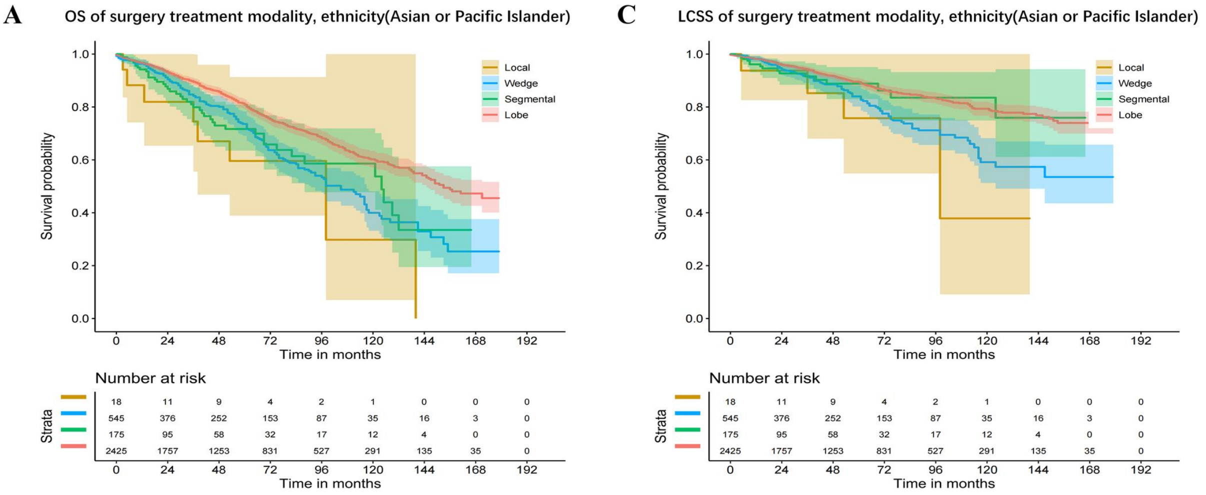

B

			Local
		Wedge	0.62(0.29,1.33)
	Segmental	1.00(0.71,1.39)	0.62(0.31,1.26)
Lobe	0.62(0.52,0.74)	0.62(0.45,0.84)	0.38(0.19,0.77)

D

			Local
		Wedge	0.69(0.25,1.88)
	Segmental	0.68(0.40,1.15)	0.47(0.16,1.40)
Lobe	0.83(0.50,1.37)	0.56(0.44,0.71)	0.38(0.14,1.03)

Figure S25 Kaplan–Meier curves of OS **(A)** and LCSS **(B)** and HR (95% CI) of OS **(C)** and LCSS **(D)** among different surgery modalities in patients with years of diagnosis (2004-2008).

A B

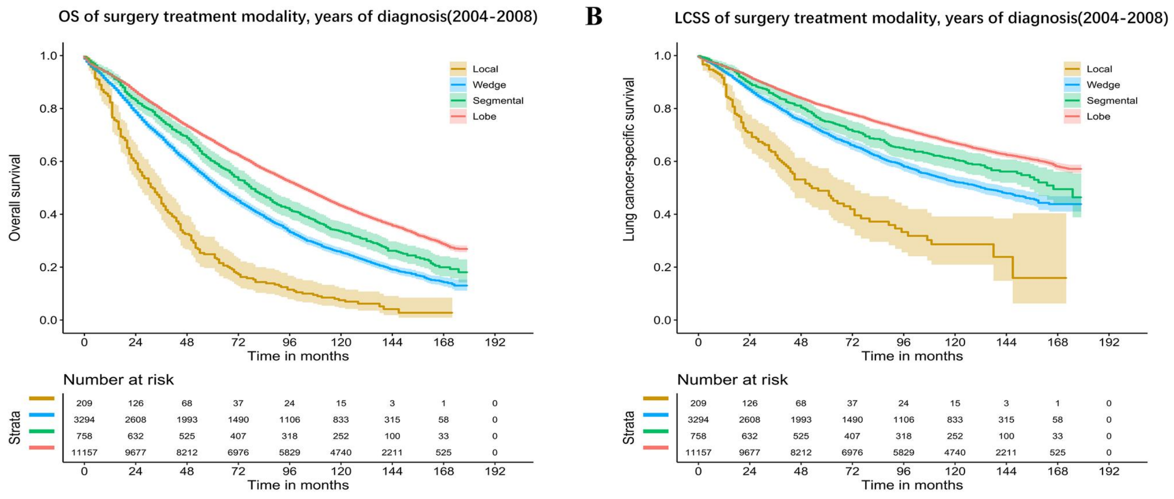

C

			Local
		Wedge	0.49(0.42,0.56)
	Segmental	0.77(0.71,0.84)	0.39(0.33,0.46)
Lobe	0.78(0.71,0.84)	0.62(0.59,0.64)	0.30(0.26,0.35)

D

			Local
		Wedge	0.47(0.38,0.57)
	Segmental	0.79(0.70,0.90)	0.37(0.30,0.47)
Lobe	0.79(,0.70,0.90)	0.63(0.59,0.67)	0.29(0.24,0.36)

Figure S26 Kaplan–Meier curves of OS **(A)** and LCSS **(B)** and HR (95% CI) of OS **(C)** and LCSS **(D)** among different surgery modalities in patients with years of diagnosis (2009-2013).

A B

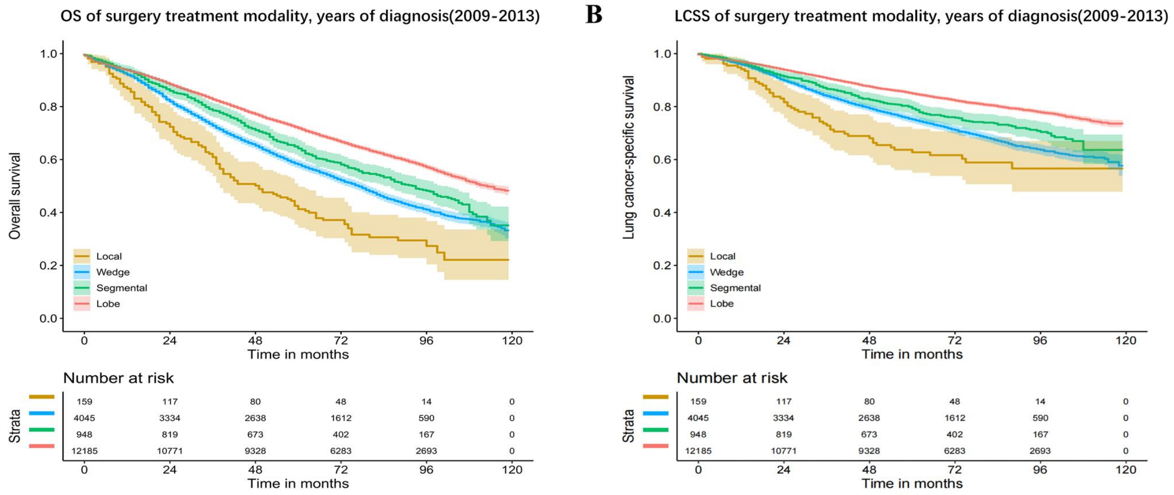

C

			Local
		Wedge	0.64(0.53,0.77)
	Segmental	0.83(0.76,0.92)	0.53(0.43,0.66)
Lobe	0.75(0.69,0.83)	0.63(0.60,0.66)	0.40(0.33,0.49)

D

			Local
		Wedge	0.68(0.52,0.89)
	Segmental	0.82(0.71,0.94)	0.55(0.41,0.74)
Lobe	0.70(0.61,0.80)	0.57(0.53,0.61)	0.39(0.30,0.50)

Figure S27 Kaplan–Meier curves of OS **(A)** and LCSS **(B)** and HR (95% CI) of OS **(C)** and LCSS **(D)** among different surgery modalities in patients with years of diagnosis (2014-2018).

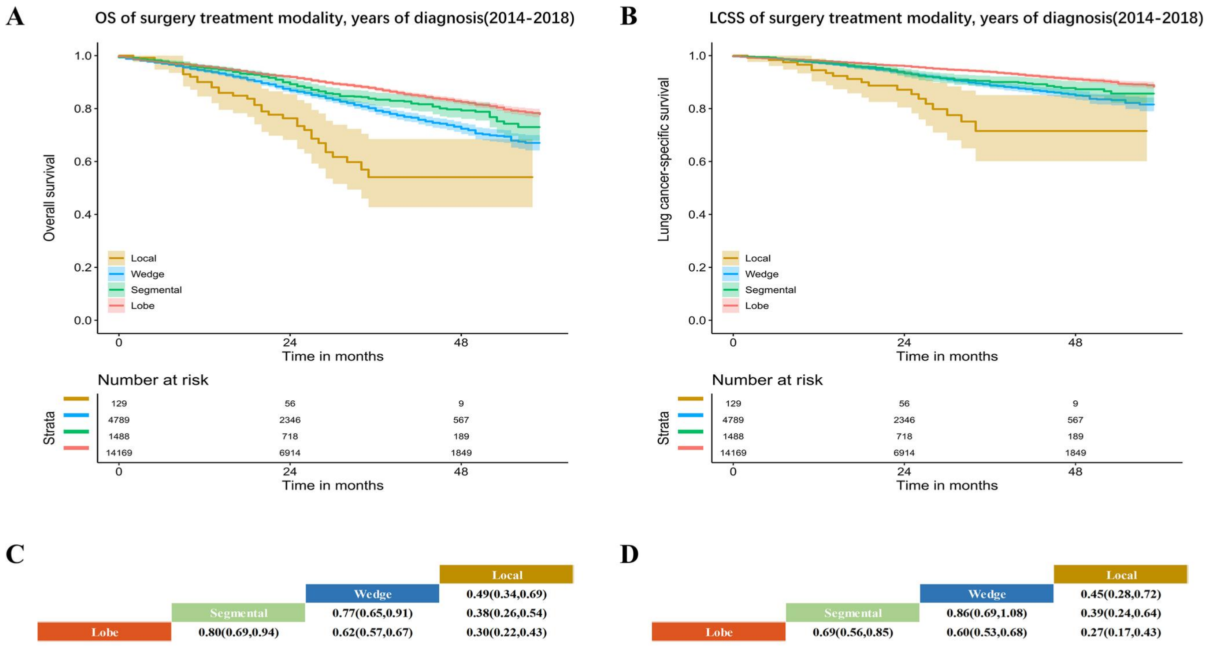

Figure S28 Kaplan–Meier curves of OS **(A)** and LCSS **(B)** and HR (95% CI) of OS **(C)** and LCSS **(D)** among different surgery modalities in patients with histologic type (LADC).

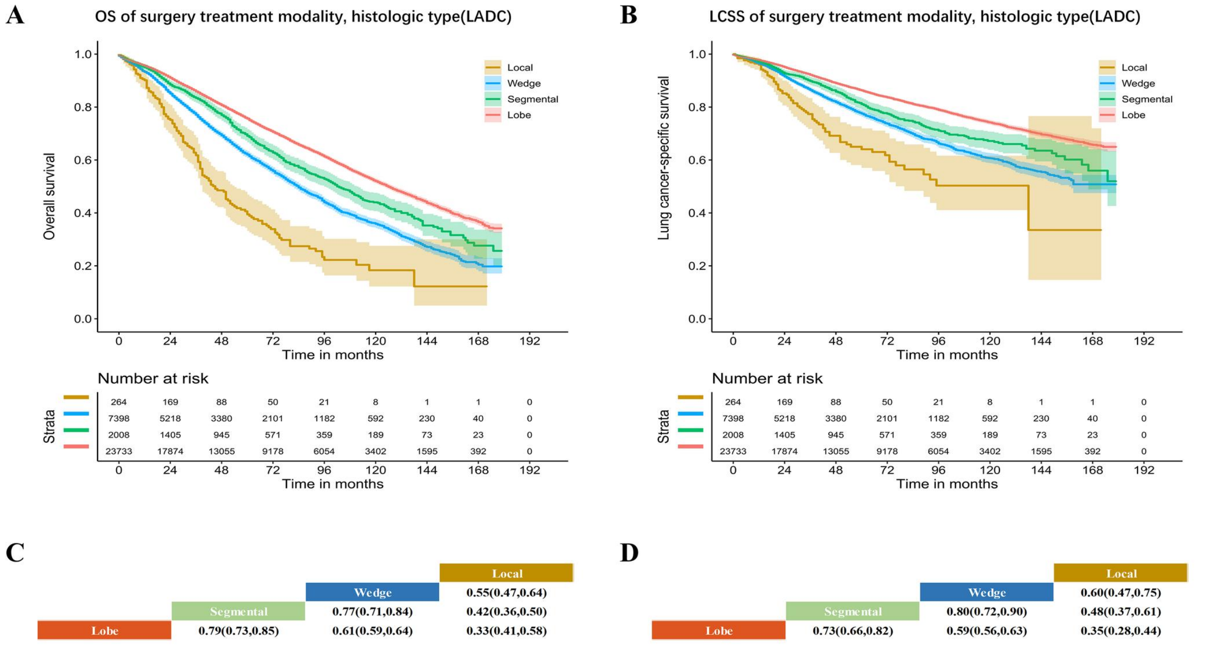

Figure S29 Kaplan–Meier curves of OS **(A)** and LCSS **(B)** and HR (95% CI) of OS **(C)** and LCSS **(D)** among different surgery modalities in patients with histologic type (LSCC).

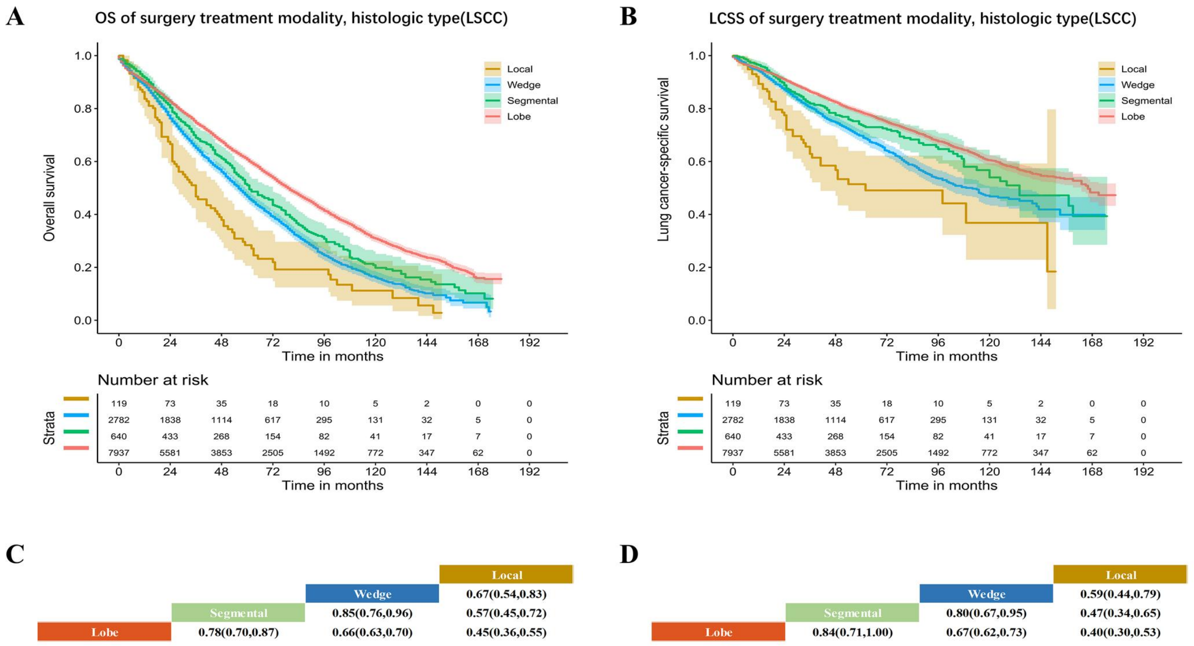

Figure S30 Kaplan–Meier curves of OS **(A)** and LCSS **(B)** and HR (95% CI) of OS **(C)** and LCSS **(D)** among different surgery modalities in patients with histologic type (others).

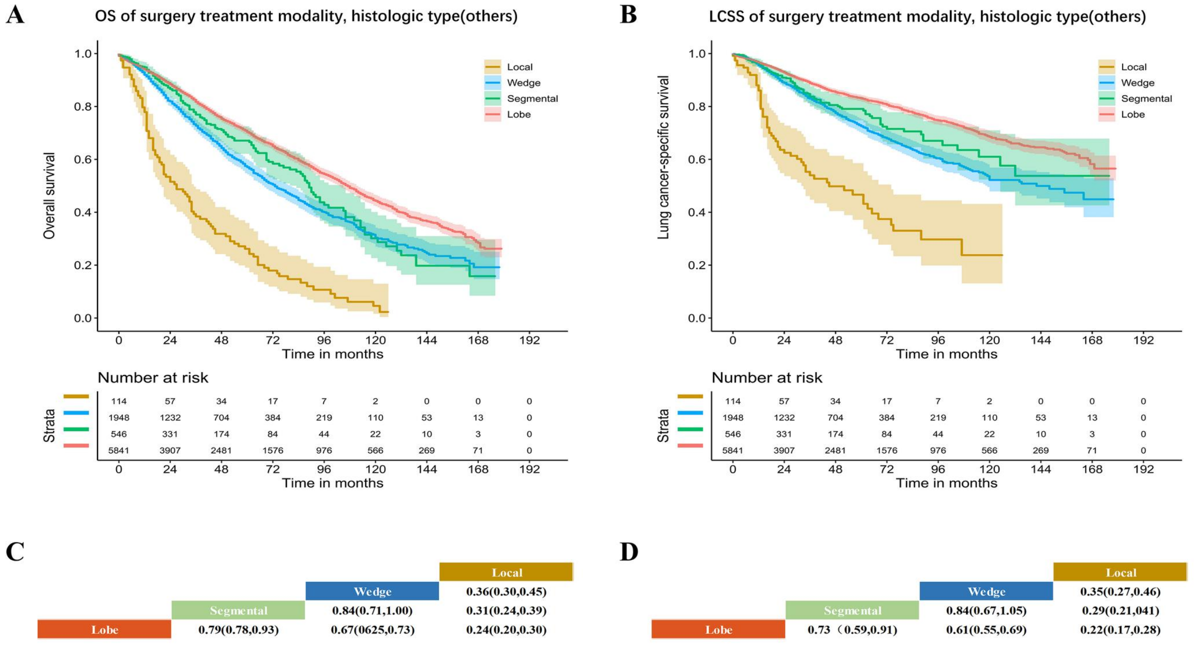

Figure S31 Kaplan–Meier curves of OS **(A)** and LCSS **(B)** and HR (95% CI) of OS **(C)** and LCSS **(D)** among different surgery modalities in patients with location (upper lobe).

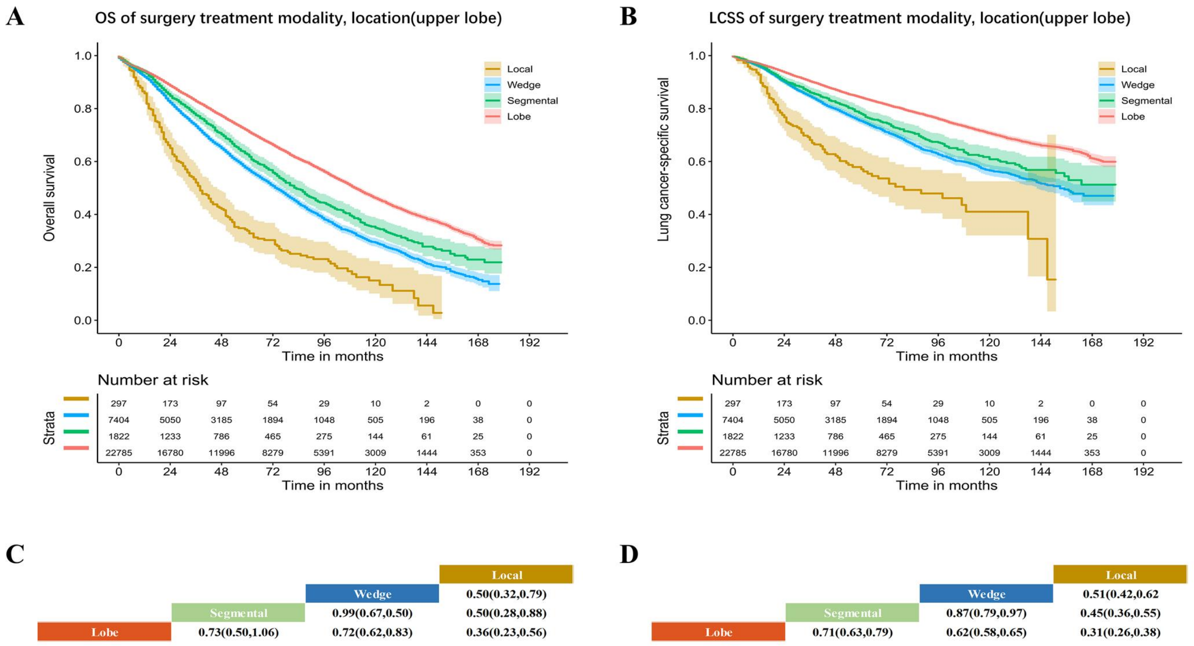

Figure S32 Kaplan–Meier curves of OS **(A)** and LCSS **(B)** and HR (95% CI) of OS **(C)** and LCSS **(D)** among different surgery modalities in patients with location (middle lobe).

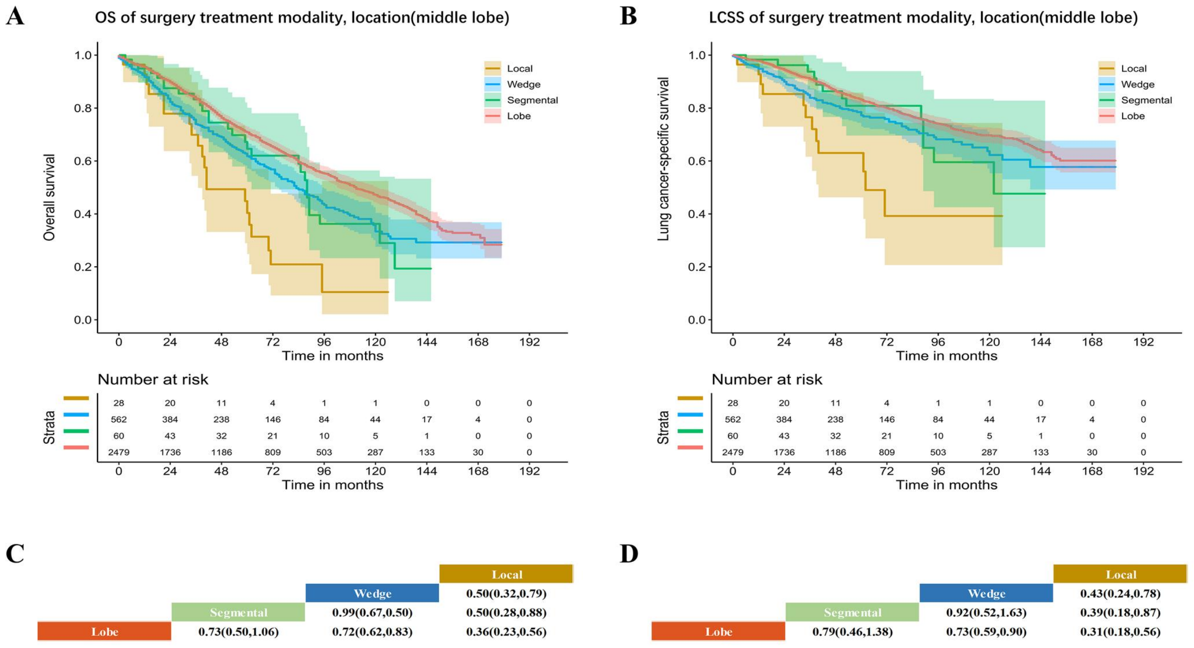

Figure S33 Kaplan–Meier curves of OS **(A)** and LCSS **(B)** and HR (95% CI) of OS **(C)** and LCSS **(D)** among different surgery modalities in patients with location (lower lobe).

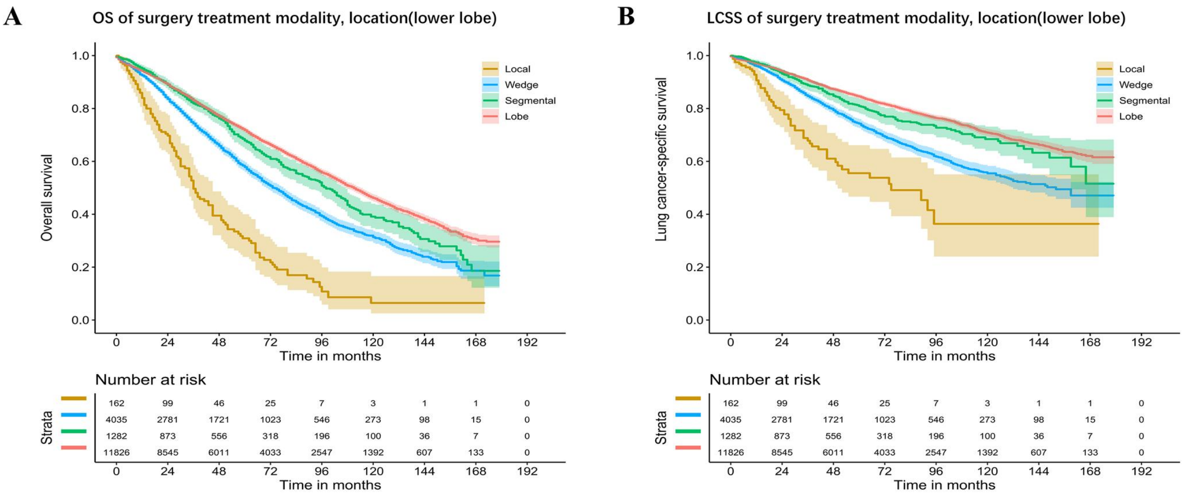

C

			Local
		Wedge	0.50(0.32,0.79)
	Segmental	0.99(0.67,0.50)	0.50(0.28,0.88)
Lobe	0.73(0.50,1.06)	0.72(0.62,0.83)	0.36(0.23,0.56)

D

			Local
		Wedge	0.49(0.38,0.64)
	Segmental	0.69(0.59,0.80)	0.34(0.25,0.45)
Lobe	0.86(0.74,0.99)	0.59(0.54,0.64)	0.29(0.22,0.38)

Figure S34 Kaplan–Meier curves of OS **(A)** and LCSS **(B)** and HR (95% CI) of OS **(C)** and LCSS **(D)** among different surgery modalities in patients with marital status (married).

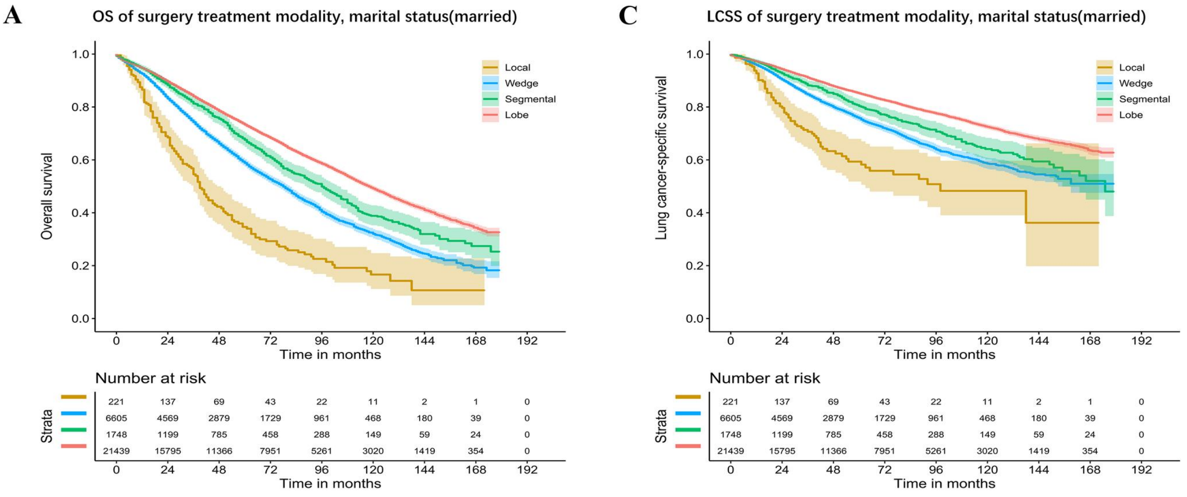

B

			Local
		Wedge	0.54(0.46,0.64)
	Segmental	0.76(0.70,0.83)	0.41(0.34,0.49)
Lobe	0.80(0.74,0.87)	0.61(0.58,0.64)	0.33(0.28,0.39)

D

			Local
		Wedge	0.55(0.44,0.70)
	Segmental	0.79(0.70,0.90)	0.44(0.34,0.57)
Lobe	0.75(0.67,0.84)	0.60(0.56,0.63)	0.33(0.26,0.42)

Figure S35 Kaplan–Meier curves of OS **(A)** and LCSS (B) and HR (95% CI) of OS **(C)** and LCSS **(D)** among different surgery modalities in patients with marital status (single).

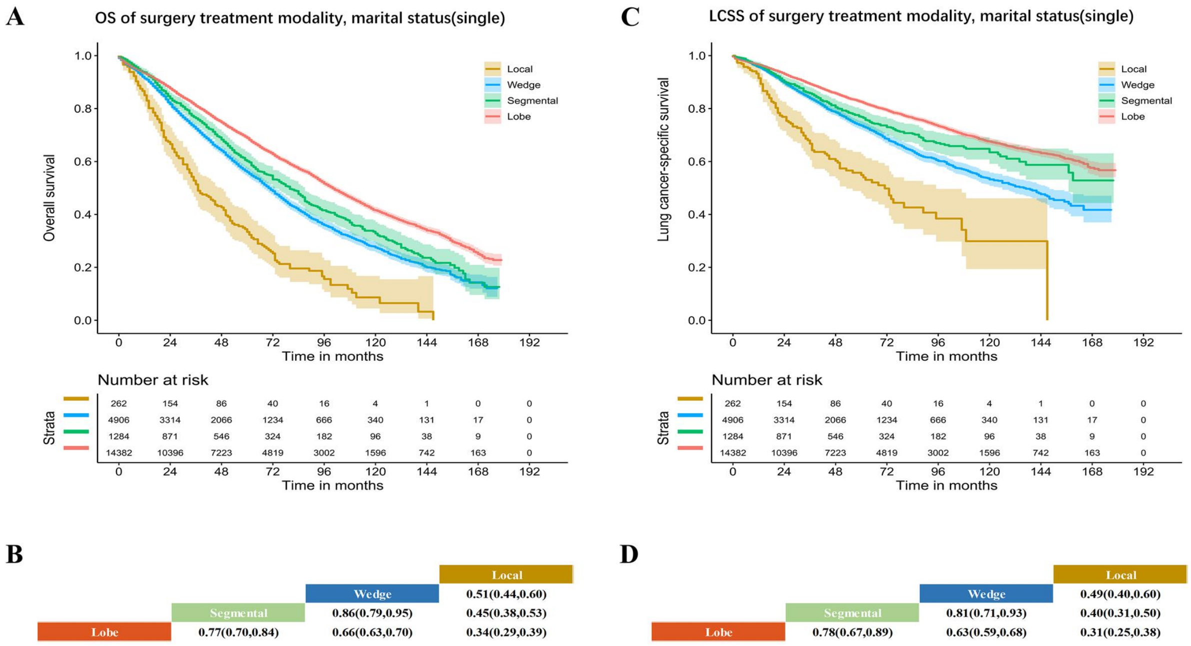

Supplement: S1 File — (PDF) [file pone.0298470.s001.pdf]
