# Supplementary material for: Treatment strategies for stage IA non-small cell lung cancer: A SEER-based population study
Source: PLoS One. 2024 Apr 29;19(4):e0298470. doi: 10.1371/journal.pone.0298470 (PMC11057715; doi:10.1371/journal.pone.0298470)
Supplement: S2 File — (PDF) [file pone.0298470.s002.pdf]

Supplementary Data – Tables

Table S1 Characteristics of patients with stage IA NSCLC.

Table S2 Characteristics of patients with stage IA NSCLC treated by multiple treatment modalities.

Table S3 Univariate and Multivariate Cox regression analysis for overall survival (OS) and lung cancer-specific survival (LCSS) in patients with IA NSCLC treated by multiple treatment modalities.

Table S4 Univariate and Multivariate Cox regression analysis for overall survival (OS) and lung cancer-specific survival (LCSS) in patients with IA NSCLC treated by surgery alone.

Table S5 Characteristics of patients with stage IA NSCLC treated by surgery alone.

Table S1 Characteristics of patients with stage IA NSCLC.

Variable	Case (%)
**Total**	**89147**
**Age**	
≤65	23330 (26.17)
>65	65817 (73.83)
**Sex**	
Female	49624 (55.67)
Male	39523 (44.33)
**Ethnicity**	
White	76056 (85.32)
Black	7739 (8.68)
Asian or Pacific Islander	4721 (5.30)
American Indian/Alaska Native	405 (0.45)
Unknown	226 (0.25)
**Years of diagnosis**	
2004-2008	23033 (25.84)
2009-2013	28283 (31.73)
2014-2018	37831 (42.44)
**Histologic type**	
LADC	50863 (57.06)
LSCC	21215 (23.80)
Others	17069 (19.15)
**Location**	
Upper lobe	53775 (60.32)
Middle lobe	4966 (5.57)
Lower lobe	28808 (32.32)
Unknown	1598(1.79)
**Surgery**	
Local[a]	581 (0.65)
Wedge	13287 (14.90)
Segmental	3389 (3.80)
Lobe	39112 (43.87)
NOS	2215 (2.48)
No	30563 (34.28)
**Chemotherapy**	
Yes	5068 (5.68)
No/unknown	84079 (94.32)
**Radiotherapy**	
Yes	22968 (25.76)
No/unknown	66179 (74.24)
**Marital status**	
Married[b]	46448 (52.10)

Single[c]	38369 (43.04)
Unknown	4330 (4.86)
**Treatment modality**	
Observation	8055 (9.04)
CA	1058 (1.19)
RA	19688 (22.08)
RC	1762 (1.98)
SA	55262 (61.99)
SC	1804 (2.02)
SR	1074 (1.20)
SRC	444 (0.50)

Abbreviations: CA: Chemotherapy alone; LADC: Lung adenocarcinoma; LSCC: Lung squamous cell cancer; NOS: not otherwise specified; RA: Radiation alone; RC: Radiation+Chemotherapy; SA: Surgery alone; SC: Surgery+Chemotherapy; SR: Surgery+Radiation; SRC: Surgery+Radiation+Chemotherapy.

[a] Local tumor destruction (includes laser ablation, cryosurgery, electrocautery and fulguration).

[b] Including marital status: married or with partner.

[c] Including marital status: single, divorced/separated or widowed.

Table S2 Characteristics of patients with stage IA NSCLC treated by multiple treatment modalities.

Variable	Observation	CA	RA	RC	SA	SC	SR	SRC	Overall Cohort N = 89147
	N (%)	N (%)	N (%)	N (%)	N (%)	N (%)	N (%)	N (%)	N (%)
**Sociodemographics**									
**Age**									
≤65	1363 (16.92)	251 (23.72)	2572 (13.06)	418 (23.72)	17495 (31.66)	787 (43.63)	261 (24.30)	183 (41.22)	23330 (26.17)
>65	6692 (83.08)	807 (76.28)	17116 (86.94)	1344 (76.28)	37767 (68.34)	1017 (56.37)	813 (75.70)	261 (58.78)	65817 (73.83)
**Sex**									
Female	4229 (52.50)	552 (52.17)	10564 (53.66)	865 (49.09)	31581 (57.15)	1035 (57.37)	562 (52.33)	236 (53.15)	49624 (55.67)
Male	3826 (47.50)	506 (47.83)	9124 (46.34)	897 (50.91)	23681 (42.85)	769 (42.63)	512 (47.67)	208 (46.85)	39523 (44.33)
**Ethnicity**									
White	6541 (81.20)	849 (80.25)	17028 (86.49)	1493 (84.73)	47280 (85.56)	1550 (85.92)	930 (86.59)	385 (86.71)	76056 (85.32)
Black	1002 (12.44)	125 (11.81)	1805 (9.17)	199 (11.29)	4313 (7.80)	145 (8.04)	110 (10.24)	40 (9.01)	7739 (8.68)
Asian or Pacific Islander	450 (5.59)	80 (7.56)	694 (3.52)	62 (3.52)	3287 (5.95)	98 (5.43)	32 (2.98)	18 (4.05)	4721 (5.30)
American Indian/Alaska Native	39 (0.48)	3 (0.28)	129 (0.66)	7 (0.40)	219 (0.40)	5 (0.28)	2 (0.19)	1 (0.23)	405 (0.45)
Unknown	23 (0.29)	1 (0.09)	32 (0.16)	1 (0.06)	163 (0.29)	6 (0.33)	0 (0.00)	0 (0.00)	226 (0.25)
**Years of diagnosis**									
2004-2008	2187 (27.15)	339 (32.04)	2188 (11.11)	593 (33.65)	16296 (29.49)	855 (47.39)	378 (35.20)	197 (44.37)	23033 (25.84)
2009-2013	2554 (31.71)	365 (34.50)	5890 (29.92)	543 (30.82)	17890 (32.37)	494 (27.38)	395 (36.78)	152 (34.23)	28283 (31.73)
2014-2018	3314 (41.14)	354 (33.46)	11610 (58.97)	626 (35.53)	21076 (38.14)	455 (25.22)	301 (28.03)	95 (21.40)	37831 (42.44)
**Histologic type**									
LADC	3721 (46.19)	565 (53.40)	9314 (47.31)	785 (44.55)	34574 (62.56)	1110 (61.53)	553 (51.49)	241 (54.28)	50863 (57.06)
LSCC	1765 (21.91)	264 (24.95)	5868 (29.80)	585 (33.20)	11945 (21.62)	333 (18.46)	338 (31.47)	117 (26.35)	21215 (23.80)
Others	2569 (31.89)	229 (21.64)	4506 (22.89)	392 (22.25)	8743 (15.82)	361 (20.01)	183 (17.04)	86 (19.37)	17069 (19.15)
**Location**									
Upper lobe	4725 (58.66)	586 (55.39)	11971 (60.80)	1100 (62.43)	33385 (60.41)	1093 (60.59)	644 (59.96)	271 (61.04)	53775 (60.32)
Middle lobe	439 (5.45)	64 (6.05)	933 (4.74)	78 (4.43)	3276 (5.93)	97 (5.38)	50 (4.66)	29 (6.53)	4966 (5.57)
Lower lobe	2558 (31.76)	365 (34.50)	6423 (32.62)	510 (28.94)	17888 (32.37)	577 (31.98)	356 (33.15)	131 (29.50)	28808 (32.32)
Unknown	333 (4.13)	43 (4.06)	361 (1.83)	74 (4.20)	713 (1.29)	37 (2.05)	24 (2.23)	13 (2.93)	1598(1.79)
**Marital status**									
Married[a]	3135 (38.92)	520 (49.15)	8944 (45.43)	911 (51.70)	31054 (56.19)	1062 (58.87)	559 (52.05)	263 (59.23)	46448 (52.10)
Single[b]	4467 (55.46)	481 (45.46)	9728 (49.41)	786 (44.61)	21610 (39.10)	663 (36.75)	466 (43.39)	168 (37.84)	38369 (43.04)
Unknown	453 (5.62)	57 (5.39)	1016 (5.16)	65 (3.69)	2598 (4.70)	79 (4.38)	49 (4.56)	13 (2.93)	4330 (4.86)

Abbreviations: CA: Chemotherapy alone; LADC: Lung adenocarcinoma; LSCC: Lung squamous cell cancer; NOS: not otherwise specified; RA: Radiation alone; RC: Radiation+Chemotherapy; SA: Surgery alone; SC: Surgery+Chemotherapy; SR: Surgery+Radiation; SRC: Surgery+Radiation+Chemotherapy.

[a] Including marital status: married or with partner.

[b] Including marital status: single, divorced/separated or widowed.

Table S3 Univariate and Multivariate Cox regression analysis for overall survival (OS) and lung cancer-specific survival (LCSS) in patients with IA NSCLC treated by multiple treatment modalities.

Variable	OS				LCSS			
	Univariate		Multivariate		Univariate		Multivariate	
	HR (95%CI)	P-value	HR (95%CI)	P-value	HR (95%CI)	P-value	HR (95%CI)	P-value
**Age**								
≤65	1.00 (Ref)		1.00 (Ref)		1.00 (Ref)		1.00 (Ref)	
>65	1.96(1.91-2.01)	<0.001	1.63(1.59-1.67)	<0.001	1.67(1.62-1.72)	<0.001	1.39(1.34-1.43)	<0.001
**Sex**								
Female	1.00 (Ref)		1.00 (Ref)		1.00 (Ref)		1.00 (Ref)	
Male	1.42(1.39-1.45)	<0.001	1.41(1.39-1.44)	<0.001	1.36(1.32-1.39)	<0.001	1.35(1.31-1.38)	<0.001
**Ethnicity**								
White	1.00 (Ref)		1.00 (Ref)		1.00 (Ref)		1.00 (Ref)	
Black	1.06(1.02-1.10)	0.001	0.98(0.95-1.01)	0.247	1.10(1.05-1.15)	<0.001	0.99(0.95-1.04)	0.709
Asian or Pacific Islander	0.64(0.61-0.68)	<0.001	0.72(0.69-0.76)	<0.001	0.68(0.63-0.73)	<0.001	0.76(0.71-0.82)	<0.001
American Indian/Alaska Native	1.11(0.97-1.28)	0.132	1.07(0.94-1.24)	0.309	1.14(0.94-1.38)	0.179	1.09(0.91-1.32)	0.349
Unknown	0.19(0.12-0.31)	<0.001	0.23(0.14-0.36)	<0.001	0.13(0.06-0.29)	<0.001	0.16(0.07-0.35)	<0.001
**Years of diagnosis**								
2004-2008	1.00 (Ref)		1.00 (Ref)		1.00 (Ref)		1.00 (Ref)	
2009-2013	0.97(0.95-0.99)	0.002	0.88(0.86-0.90)	<0.001	0.89(0.86-0.91)	<0.001	0.82(0.80-0.85)	<0.001
2014-2018	0.83(0.80-0.85)	<0.001	0.70(0.68-0.72)	<0.001	0.71(0.68-0.73)	<0.001	0.61(0.59-0.64)	<0.001
**Histologic type**								
LADC	1.00 (Ref)		1.00 (Ref)		1.00 (Ref)		1.00 (Ref)	
LSCC	1.74(0.1.70-1.78)	<0.001	1.45(1.42-1.48)	<0.001	1.65(1.60-1.70)	<0.001	1.39(1.35-1.43)	<0.001
Others	1.62(1.58-1.66)	<0.001	1.23(1.20-1.26)	<0.001	1.59(1.54-1.65)	<0.001	1.19(1.15-1.23)	<0.001
**Location**								
Upper lobe	1.00 (Ref)		1.00 (Ref)		1.00 (Ref)			
Middle lobe	0.92(0.88-0.96)	<0.001	0.99(0.95-1.03)	0.649	0.95(0.90-1.02)	0.153		
Lower lobe	0.99(0.97-1.01)	0.466	1.00(0.98-1.02)	0.714	0.99(0.97-1.02)	0.670		
Unknown	1.40(1.31-1.49)	<0.001	1.12(1.05-1.19)	0.001	1.55(1.42-1.69)	<0.001		
**Marital status**								
Married[a]	1.00 (Ref)		1.00 (Ref)		1.00 (Ref)		1.00 (Ref)	
Single[b]	1.29(1.26-1.31)	<0.001	1.24(1.21-1.26)	<0.001	1.29(1.26-1.33)	<0.001	1.21(1.18-1.25)	<0.001
Unkown	1.11(1.06-1.17)	<0.001	1.08(1.03-1.14)	0.001	1.03(0.96-1.1)	0.452	1.00(0.93-1.07)	0.997

**Treatment modality**								
Observation	1.00 (Ref)		1.00 (Ref)		1.00 (Ref)		1.00 (Ref)	
CA	0.91(0.85-0.98)	0.012	0.94(0.88-1.01)	0.109	1.11(1.02-1.21)	0.016	1.13(1.04-1.24)	0.005
RA	0.56(0.54-0.58)	<0.001	0.57(0.55-0.58)	<0.001	0.49(0.47-0.51)	<0.001	0.51(0.49-0.53)	<0.001
RC	0.72(0.68-0.76)	<0.001	0.70(0.66-0.74)	<0.001	0.87(0.81-0.94)	<0.001	0.84(0.78-0.90)	<0.001
SA	0.20(0.20-0.21)	<0.001	0.23(0.22-0.23)	<0.001	0.18(0.17-0.19)	<0.001	0.20(0.19-0.20)	<0.001
SC	0.25(0.24-0.27)	<0.001	0.29(0.27-0.31)	<0.001	0.32(0.30-0.35)	<0.001	0.35(0.32-0.38)	<0.001
SR	0.42(0.39-0.45)	<0.001	0.41(0.38-0.45)	<0.001	0.45(0.41-0.50)	<0.001	0.44(0.40-0.49)	<0.001
SRC	0.46(0.41-0.52)	<0.001	0.51(0.45-0.57)	<0.001	0.59(0.52-0.68)	<0.001	0.62(0.54-0.70)	<0.001

Abbreviations: CA: Chemotherapy alone; CI: confidence interval; HR: hazard ratio; OS: Overall survival; LADC: Lung adenocarcinoma; LCSS: Lung cancer-specific survival; LSCC: Lung squamous cell cancer; RA: Radiation alone; RC: Radiation+Chemotherapy; SA: Surgery alone; SC: Surgery+Chemotherapy; SR: Surgery+Radiation; SRC: Surgery+Radiation+Chemotherapy.

[a] Including marital status: married or with partner.

[b] Including marital status: single, divorced/separated or widowed.

Table S4 Univariate and Multivariate Cox regression analysis for overall survival (OS) and lung cancer-specific survival (LCSS) in patients with IA NSCLC treated by surgery alone.

Variable	OS				LCSS			
	Univariate		Multivariate		Univariate		Multivariate	
	HR (95%CI)	P-value	HR (95%CI)	P-value	HR (95%CI)	P-value	HR (95%CI)	P-value
**Age**								
≤65	1.00 (Ref)		1.00 (Ref)		1.00 (Ref)		1.00 (Ref)	
>65	1.91(1.85-1.97)	<0.001	1.78(1.72-1.84)	<0.001	1.57(1.50-1.64)	<0.001	1.47(1.40-1.54)	<0.001
**Sex**								
Female	1.00 (Ref)		1.00 (Ref)		1.00 (Ref)		1.00 (Ref)	
Male	1.49(1.45-1.53)	<0.001	1.50(1.46-1.54)	<0.001	1.42(1.36-1.47)	<0.001	1.42(1.37-1.48)	<0.001
**Ethnicity**								
White	1.00 (Ref)		1.00 (Ref)		1.00 (Ref)		1.00 (Ref)	
Black	0.96(0.91-1.01)	0.116	1.00(0.95-1.05)	0.852	1.01(0.94-1.08)	0.822	1.03(0.96-1.11)	0.412
Asian or Pacific Islander	0.62(0.58-0.66)	<0.001	0.70(0.65-0.75)	<0.001	0.64(0.58-0.71)	<0.001	0.72(0.65-0.80)	<0.001
American Indian/Alaska Native	0.95(0.76-1.18)	0.627	1.06(0.85-1.32)	0.593	0.88(0.63-1.22)	0.441	0.96(0.69-1.33)	0.789
Unknown	0.10(0.04-0.25)	<0.001	0.13(0.05-0.31)	<0.001	0.04(0.01-0.30)	0.002	0.05(0.01-0.38)	0.003
**Years of diagnosis**								
2004-2008	1.00 (Ref)		1.00 (Ref)		1.00 (Ref)		1.00 (Ref)	
2009-2013	0.86(0.83-0.89)	<0.001	0.86(0.84-0.89)	<0.001	0.79(0.76-0.83)	<0.001	0.80(0.77-0.84)	<0.001
2014-2018	0.61(0.59-0.64)	<0.001	0.63(0.60-0.66)	<0.001	0.54(0.50-0.57)	<0.001	0.55(0.51-0.58)	<0.001
**Histologic type**								
LADC	1.00 (Ref)		1.00 (Ref)		1.00 (Ref)		1.00 (Ref)	
LSCC	1.78(1.73-1.83)	<0.001	1.53(1.49-1.58)	<0.001	1.62(1.55-1.69)	<0.001	1.42(1.36-1.49)	<0.001
Others	1.25(1.20-1.30)	<0.001	1.22(1.17-1.26)	<0.001	1.30(1.23-1.37)	<0.001	1.27(1.20-1.34)	<0.001
**Location**								
Upper lobe	1.00 (Ref)		1.00 (Ref)		1.00 (Ref)			
Middle lobe	0.94(0.88-1.00)	0.034	1.01(0.95-1.07)	0.789	0.98(0.90-1.06)	0.644		
Lower lobe	0.99(0.96-1.02)	0.416	0.99(0.96-1.02)	0.583	1.00(0.96-1.04)	0.860		
Unknown	1.12(1.00-1.26)	0.055	1.11(0.99-1.24)	0.08	1.18(1.01-1.39)	0.042		
**Marital status**								
Married[a]	1.00 (Ref)		1.00 (Ref)		1.00 (Ref)		1.00 (Ref)	
Single[b]	1.22(1.18-1.25)	<0.001	1.29(1.25-1.33)	<0.001	1.20(1.15-1.25)	<0.001	1.25(1.20-1.31)	<0.001
Unknown	1.03(0.96-1.11)	0.357	1.12(1.04-1.20)	0.002	0.92(0.83-1.02)	0.12	0.99(0.89-1.10)	0.898

**Surgery modality**								
Local*	1.00 (Ref)		1.00 (Ref)		1.00 (Ref)		1.00 (Ref)	
Wedge	0.52(0.46-0.58)	<0.001	0.59(0.53-0.66)	<0.001	0.50(0.43-0.59)	<0.001	0.58(0.50-0.67)	<0.001
Segmental	0.41(0.37-0.46)	<0.001	0.49(0.43-0.55)	<0.001	0.40(0.34-0.48)	<0.001	0.48(0.40-0.56)	<0.001
Lobe	0.32(0.29-0.36)	<0.001	0.39(0.35-0.43)	<0.001	0.31(0.27-0.36)	<0.001	0.36(0.31-0.42)	<0.001

Abbreviations: CI: confidence interval; HR: hazard ratio; OS: Overall survival; LADC: Lung adenocarcinoma; LCSS: Lung cancer-specific survival; LSCC: Lung squamous cell cancer.

[a] Including marital status: married or with partner.

[b] Including marital status: single, divorced/separated or widowed.

[c] Local tumor destruction (includes laser ablation, cryosurgery, electrocautery and fulguration).

Table S5 Characteristics of patients with stage IA NSCLC treated by surgery alone.

Variable	Local*	Wedge	Segmental	Lobe	NOS	Overall Cohort N = 55262
	N (%)	N (%)	N (%)	N (%)	N (%)	N (%)
**Age**						
≤65	81 (16.30)	3153 (26.00)	833 (26.08)	12714 (33.89)	714 (36.96)	17495 (31.66)
>65	416 (83.70)	8975 (74.00)	2361 (73.92)	24797 (66.11)	1218 (63.04)	37767 (68.34)
**Sex**						
Female	278 (55.94)	6944 (57.26)	1936 (60.61)	21403 (57.06)	1020 (52.80)	31581 (57.15)
Male	219 (44.06)	5184 (42.74)	1258 (39.39)	16108 (42.94)	912 (47.20)	23681 (42.85)
**Ethnicity**						
White	443 (89.13)	10534 (86.86)	2773 (86.82)	31905 (85.06)	1625 (84.11)	47280 (85.56)
Black	35 (7.04)	962 (7.93)	228 (7.14)	2912 (7.76)	176 (9.11)	4313 (7.80)
Asian or Pacific Islander	18 (3.62)	545 (4.49)	175 (5.48)	2425 (6.46)	124 (6.42)	3287 (5.95)
American Indian/Alaska Native	1 (0.20)	48 (0.40)	8 (0.25)	158 (0.42)	4 (0.21)	219 (0.40)
Unknown	0 (0.00)	39 (0.32)	10 (0.31)	111 (0.30)	3 (0.16)	163 (0.29)
**Years of diagnosis**						
2004-2008	209 (42.05)	3294 (27.16)	758 (23.73)	11157 (29.74)	878 (45.45)	16296 (29.49)
2009-2013	159 (31.99)	4045 (33.35)	948 (29.68)	12185 (32.48)	553 (28.62)	17890 (32.37)
2014-2018	129 (25.96)	4789 (39.49)	1488 (46.59)	14169 (37.77)	501 (25.93)	21076 (38.14)
**Histologic type**						
LADC	264 (53.12)	7398 (61.00)	2008 (62.87)	23733 (63.27)	1171 (60.61)	34574 (62.56)
LSCC	119 (23.94)	2782 (22.94)	640 (20.04)	7937 (21.16)	467 (24.17)	11945 (21.62)
Others	114 (22.94)	1948 (16.06)	546 (17.09)	5841 (15.57)	294 (15.22)	8743 (15.82)
**Location**						
Upper lobe	297 (59.76)	7404 (61.05)	1822 (57.04)	22785 (60.74)	1077 (55.75)	33385 (60.41)
Middle lobe	28 (5.63)	562 (4.63)	60 (1.88)	2479 (6.61)	147 (7.61)	3276 (5.93)
Lower lobe	162 (32.60)	4035 (33.27)	1282 (40.14)	11826 (31.53)	583 (30.18)	17888 (32.37)
Unknown	10 (2.01)	127 (1.05)	30 (0.94)	421 (1.12)	125 (6.47)	713 (1.29)
**Marital status**						
Married[a]	221 (44.47)	6605 (54.46)	1748 (54.73)	21439 (57.15)	1041 (53.88)	31054 (56.19)
Single[b]	262 (52.72)	4906 (40.45)	1284 (40.20)	14382 (38.34)	776 (40.17)	21610 (39.10)
Unknown	14 (2.82)	617 (5.09)	162 (5.07)	1690 (4.51)	115 (5.95)	2598 (4.70)

Abbreviations: LADC: Lung adenocarcinoma; LSCC: Lung squamous cell cancer; NOS: not otherwise specified.

[a] Local tumor destruction (includes laser ablation, cryosurgery, electrocautery and fulguration);

[b] Including marital status: married or with partner;

[c] Including marital status: single, divorced/separated or widowed.
